# Transgenic cotton expressing Cry10Aa toxin confers high resistance to the cotton boll weevil

**DOI:** 10.1111/pbi.12694

**Published:** 2017-03-02

**Authors:** Thuanne Pires Ribeiro, Fabricio Barbosa Monteiro Arraes, Isabela Tristan Lourenço‐Tessutti, Marilia Santos Silva, Maria Eugênia Lisei‐de‐Sá, Wagner Alexandre Lucena, Leonardo Lima Pepino Macedo, Janaina Nascimento Lima, Regina Maria Santos Amorim, Sinara Artico, Márcio Alves‐Ferreira, Maria Cristina Mattar Silva, Maria Fatima Grossi‐de‐Sa

**Affiliations:** ^1^ Brasilia Federal University (UnB) Brasília DF Brazil; ^2^ Embrapa Genetic Resources and Biotechnology Brasília DF Brazil; ^3^ Rio Grande do Sul Federal University Porto Alegre RS Brazil; ^4^ Agricultural Research Company of Minas Gerais State Uberaba MG Brazil; ^5^ Embrapa Cotton Campina Grande PB Brazil; ^6^ Rio de Janeiro Federal University Rio de Janeiro RJ Brazil; ^7^ Catholic University of Brasilia Brasília DF Brazil

**Keywords:** *Gossypium hirsutum*, genetic transformation, *Anthonomus grandis*, insect pest, plant resistance, *Bt* toxin

## Abstract

Genetically modified (GM) cotton plants that effectively control cotton boll weevil (CBW), which is the most destructive cotton insect pest in South America, are reported here for the first time. This work presents the successful development of a new GM cotton with high resistance to CBW conferred by Cry10Aa toxin, a protein encoded by entomopathogenic *Bacillus thuringiensis* (*Bt*) gene. The plant transformation vector harbouring *cry10Aa* gene driven by the cotton ubiquitination‐related promoter *uce*A1.7 was introduced into a Brazilian cotton cultivar by biolistic transformation. Quantitative PCR (qPCR) assays revealed high transcription levels of *cry10Aa* in both T_0_ GM cotton leaf and flower bud tissues. Southern blot and qPCR‐based 2^−ΔΔCt^ analyses revealed that T_0_ GM plants had either one or two transgene copies. Quantitative and qualitative analyses of Cry10Aa protein expression showed variable protein expression levels in both flower buds and leaves tissues of T_0_ GM cotton plants, ranging from approximately 3.0 to 14.0 μg g^−1^ fresh tissue. CBW susceptibility bioassays, performed by feeding adults and larvae with T_0_ GM cotton leaves and flower buds, respectively, demonstrated a significant entomotoxic effect and a high level of CBW mortality (up to 100%). Molecular analysis revealed that transgene stability and entomotoxic effect to CBW were maintained in T_1_ generation as the Cry10Aa toxin expression levels remained high in both tissues, ranging from 4.05 to 19.57 μg g^−1^ fresh tissue, and the CBW mortality rate remained around 100%. In conclusion, these Cry10Aa GM cotton plants represent a great advance in the control of the devastating CBW insect pest and can substantially impact cotton agribusiness.

## Introduction

Cotton (*Gossypium hirsutum*) production is highly influenced by a large number of insect pests, and a major pest in the Americas is the cotton boll weevil (CBW) *Anthonomus grandis* (Coleoptera: Curculionidae), which causes significant losses to cotton production and impacts fiber quality (Azambuja and Degrande, 2014; Bastos *et al*., 2005; De Lima *et al*., 2013; Gallo *et al*., 2002; Habib and Fernandes, 1983; Instituto Mato‐grossense do Algodão, 2015; Ribeiro *et al*., 2010; Soria *et al*., 2013). The endophytic habit of CBW larvae into cotton reproductive structures can result in crop losses of up to 100%, especially because chemical control is only applicable during the adult weevil stage, when it feeds on immature cotton bolls (Busoli and Michelotto, 2005; Ribeiro *et al*., 2015).

*Bacillus thuringiensis* (*Bt*) has contributed to insect pest control since the 1960s (Lacey *et al*., 2001). There are currently more than 750 characterized *Bt*‐encoded entomotoxic crystal proteins (Cry), which are grouped into at least 74 different classes and are collectively active against insects, nematodes, mites and protozoans (Crickmore *et al*., 1998). Although substantial knowledge on the direct use of *Bt* for the biological control of insects has accumulated over the last decades, its commercial application is limited due to high production costs and instability of the Cry proteins under field conditions (Navon, 2000, 2013).

The broad adoption of genetically modified (GM) *Bt* cotton technology by the world's largest cotton producers, such as China, India and Brazil, has notably brought great economic benefits to producers (James, 2015). Concerning hemipteran insect control, a recent study showed that a *Bt* toxin variant (Cry51Aa2.834_16) could reduce populations of *Lygus* spp. in whole‐GM cotton plants evaluated in caged‐field trials (Gowda *et al*., 2016). Although various characterized Cry toxins are active against lepidopteran insects, far fewer Cry proteins present toxicity to coleopteran species (Donovan *et al*., 1992; James, 2015; Maagd, 2015; Pathak *et al*., 2015; Shah *et al*., 2016). Some Cry proteins, such as Cry1Ba6, Cry8Ka and Cry1Ia12, have recently been described as somewhat entomotoxic against the coleopteran pest *A. grandis* (Aguiar *et al*., 2012; Grossi‐de‐Sa *et al*., 2007; Martins *et al*., 2010; Oliveira *et al*., 2011, 2016; Silva *et al*., 2015). In this way, Cry‐independent technologies could be used as effective alternatives against coleopteran insect pest, such as recently described to control the hemipteran *Bemisia tabaci* (whitefly). Shukla *et al*. showed that the transgenic expression of an insecticidal fern protein (Tma12) with chitinase activity can protect cotton against whitefly achieving complete resistance up to T_4_ generation in contained field trials (Shukla *et al*., 2016). Unfortunately, the development of such technologies is still a challenge as, unlike Cry toxins, few molecules have cytotoxic effects only on target insect populations.

A Cry10 protein was identified in a *B. thuringiensis* subsp. *israelensis* (*Bti*) strain as being toxic to the coleopteran pest coffee berry borer (*Hypothenemus hampei*) (Méndez‐López *et al*., 2003). Later, Aguiar *et al*. (2012) demonstrated a *in vitro* specific and high activity of Cry10Aa towards CBW. Due to the biotechnological potential presented by the Cry10Aa toxin against CBW, a Brazilian cotton cultivar (BRS 372) was transformed with the *cry10Aa* gene under the control of the cotton ubiquitination‐related promoter *uce*A1.7, which provided constitutive high transgene expression levels of the entomotoxin. The expression of the Cry10Aa protein in such GM cotton plants resulted in a strong entomotoxic effect against CBW. We herein describe the first GM cotton plants with the potential to effectively control the CBW, insect pest with the greatest impact on cotton crop.

## Results

### Successful generation of Cry10Aa T_0_ GM cotton plants

The *in silico* prediction of the Cry10Aa three‐dimensional model (Figures S1, S2) and the *in vitro* revaluation of its toxicity (Table S1) are important analyses for designing an optimized DNA transformation cassette. Furthermore, the choice of a suitable promoter is crucial for toxin production at desirable levels (Figure S3). These preliminary results reveal that Cry10Aa toxin has a Cry typical 3D‐deltaendotoxin (three‐domain) conformation, which is typical of pore‐forming toxins, with seven helixes in domain I, three beta sheets in domain II and a beta sandwich in domain III (Figures S1, S2), and presents LC_50_ 6.35 μg mL^−1^ against the CBW, which indicates a similar potency of Cry10Aa to the same insect found by Aguiar *et al*. (2012) (Table S1). A comparison of tissue specificity between the *CaMV35S* and *uce*A1.7 promoters in *A. thaliana* revealed that *uce*A1.7 is probably stronger and more specific than *CaMV*35S in cotton fruit, which is the main plant organ targeted by the CBW. This data makes this promoter a better choice to drive *cry10Aa* gene expression in GM cotton plants (Figure S3).

**Figure 1:**
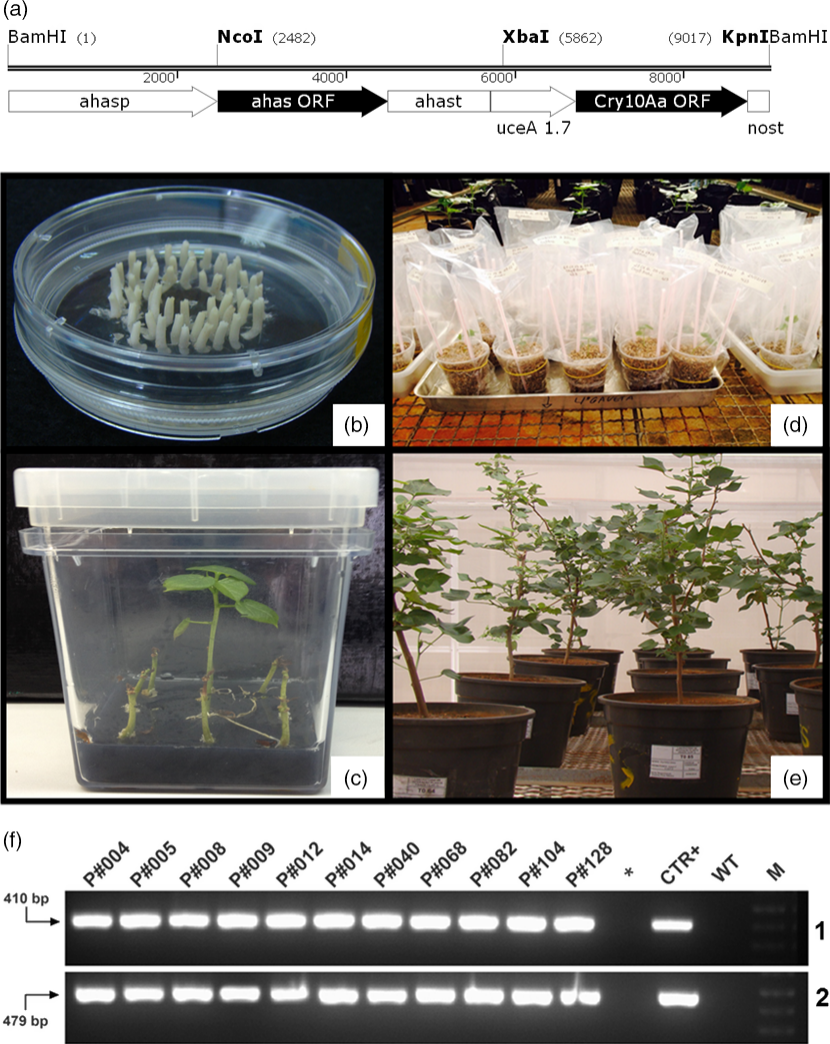
Biolistic cotton transformation with the Cry10Aa expression cassette and PCR amplification selection of cotton transformants. (a) Schematic diagram of the Cry10Aa expression cassette *uce*A1.7::*cry10Aa*‐*nos*t‐*ahas* used for cotton embryo transformation via particle bombardment; (b) Cotton embrionary axes prepared for apical meristem bombardment with DNA transformation Cry10Aa expression cassette; (c) *in vitro* selection of GM cotton plants on medium containing the selection agent Imazapyr herbicide. The *ahas* selection marker, present in the Cry10Aa expression cassette, confers resistance to imidazolinone herbicides, such as Imazapyr; (d) acclimation of Cry10Aa T_0_ GM cotton plants under greenhouse conditions, using vermiculite as a substrate; (e) acclimation of Cry10Aa T_0_ GM cotton plants under greenhouse conditions, using soil as a substrate (plants 1 year old); (f) detection of Cry10Aa T_0_ GM cotton plants via PCR. Genomic DNA was extracted from the leaves of selected plants to amplify (1) the *cry10Aa* gene (410‐bp amplicon) and (2) the *ahas* gene (479‐bp amplicon), which are both present in the Cry10Aa expression cassette used for cotton transformation. (*) asterisk represents an empty well; *ahas*,* Arabidopsis thaliana* acetohydroxyacid synthase (*ahas*) selection marker sequence that confers resistance to imidazolinone herbicides; *ahas*p, *ahas* promoter; *ahas*t, *ahas* terminator; *cry10Aa*, gene encoding the Cry10Aa toxin from the *Bacillus thuringiensis* S1804 strain; CTR+, positive control (PCR template is the original Cry10Aa expression cassette used for cotton transformation); *nos*t, nopaline synthase (*nos*) polyadenylation signal terminator; M, 1.0‐kb ladder (INVITROGEN^®^ Cat. # 10787018); WT, wild‐type non‐GM plants.

**Figure 2:**
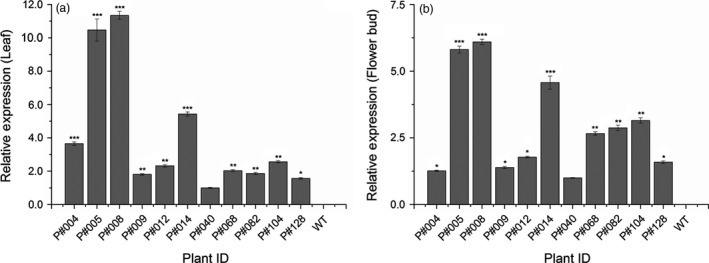
*cry10Aa* transcript relative expression as measured by quantitative PCR (qPCR) in Cry10Aa T_0_ GM cotton plants. *cry10Aa* transcript relative expression in the leaves (a) and flower buds (b) of eleven T_0_ selected cotton plants. The asterisks represent the level of statistical significance (Student's *t*‐test): (*) *P *≤ 0.05; (**) 0.05 < *P *≤ 0.01; (***) 0.01 < *P *≤ 0.001. After normalization based on the expression of plant endogenous genes, the values were plotted relative to the lowest expression value (excluding the wild‐type plants).—P#040 set as expression level 1 (one). WT, wild‐type non‐GM plants.

The Cry10Aa expression cassette for cotton transformation *uce*A1.7::*cry10Aa*‐*nos*t‐*ahas* (Figure 1a) was used to bombard 5479 cotton embrionary axes (Figure 1b), from which 151 cotton plantlets (Figure 1c) were selected under tolerance to imazapyr conferred by the *ahas* gene product. After greenhouse acclimation (Figure 1d, e), PCR analysis confirmed the insertion of the Cry10Aa expression cassette into the genome of 30 cotton transformant plants (Figure 1e, f). The PCR amplification of 410 and 479 bp fragments indicated the insertion of the *cry10Aa* and *ahas* transgenes, respectively (Figure 1f‐1, f‐2), into the genome of the bombarded cotton plants. As expected, no amplification was detected in non‐transformated plants [wild type (WT)]. This result indicates a transformation efficiency ratio of approximately 0.5%. All T_0_ plants were fertile and exhibited normal growth and phenotypes compared to those of WT cotton plants (Figures 1e, S4).

**Figure 3:**
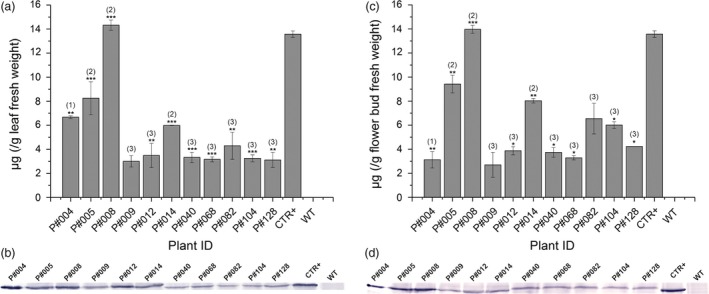
Cry10Aa protein immunodetection in Cry10Aa T_0_ GM cotton plants. (a and c) Indirect ELISA quantification and (b and d) Western blot immunodetection of Cry10Aa protein expression in the leaves (a and b) and flower buds (c and d) from Cry10Aa T_0_ GM cotton plants. The primary antibody used in all experiments was the polyclonal anti‐Cry10Aa (see Material and Methods for details). The Cry10Aa molecular weight observed in the Western blots was 77.7 kDa. In indirect ELISA and Western blot experiments, the total protein amount were 1.0 and 20.0 μg per well, respectively. The asterisks represent the level of statistical significance (Student's *t*‐test): (*) *P *≤ 0.05; (**) 0.05 < *P *≤ 0.01; (***) 0.01 < *P *≤ 0.001. Expression values of each sample were compared with the control, which was the one with the lowest expression level (excluding wild‐type plants). GM cotton plants were grouped according to the number of copies of the *cry10Aa* transgene in the genome and the protein expression level: (1) high Cry10Aa expression level with one transgene copy; (2) high Cry10Aa expression level with two transgene copies; (3) intermediate Cry10Aa expression level with two transgene copies. This classification was in accordance with Turkey's statistical test. CTR+, positive control of recombinant Cry10Aa protein purified of *Escherichia coli*; WT, wild‐type non‐GM plants.

Among the 30 PCR‐positive T_0_ GM cotton plants, only 11 plants presented a significant *cry10Aa* transgene expression level, as measured by qPCR (*cry10Aa* transcript) and ELISA (Cry10Aa protein), which were confirmed by Western blot analysis of both leaf and floral bud tissues (Figures 2, 3). The qPCR results revealed that the *cry10Aa* transgene transcript was expressed in the 11 plants in both leaves (Figure 2a; Table S2) and flower buds (Figure 2b; Table S2). The *cry10Aa* transcript expression levels in both tissues varied among GM cotton plants, although a pattern was observed: a high relative expression in leaves was invariably accompanied by a high relative expression in flower buds within the same GM plant. The same pattern was also observed for low relative expression levels (Figure 2). Notably, the P#008 cotton line presented the highest *cry10Aa* transcript expression level in both leaves and flower buds, whereas the P#040 cotton line presented the lowest levels in both tissues among all 11 cotton transforming plants (Figure 2). The top four GM plants in terms of *cry10Aa* transcript expression level were P#004, P#005, P#008 and P#014, which presented the highest *cry10Aa* transcript expression levels among the 11 cotton transforming plants, ranging from 3.5‐ to 11.0‐fold in leaves (Figure 2a) and 4.2‐ to 5.6‐fold in flower buds (Figure 2b).

ELISA and Western blot analysis using total protein extracts from leaves and flower buds indicated different levels of Cry10Aa protein production among the 11 GM cotton plants (Figure 3). ELISA revealed that Cry10Aa GM plants could express high toxin levels in both leaves and flower buds, ranging from 3.1 to 14 μg g^−1^ leaf fresh weight (Figure 3a) and 3.1–13.9 μg g^−1^ flower bud fresh weight (Figure 3c). The Western blot analysis of total extract from both tissues showed the presence of the Cry10Aa protein with approximately 77 kDa (Figure 3b, d) that was not present in the extracts of WT plants.

**Figure 4:**
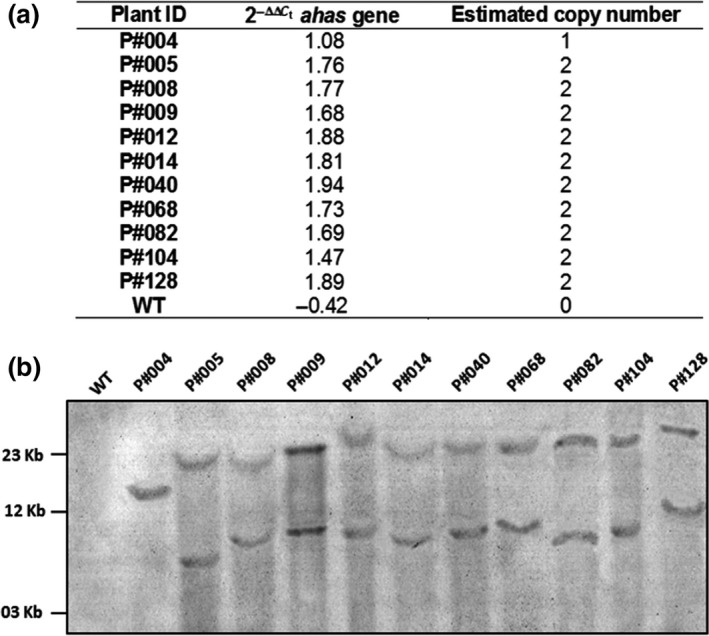
Copy number of Cry10Aa expression cassette in Cry10Aa T_0_ GM cotton plants (a) Quantitative PCR (qPCR)‐based 2^−ΔΔCt^ method, once the *ahas* transgene detected by qPCR is present in the Cry10Aa expression cassette; (b) Southern blot analysis. The genomic DNA of eleven independent Cry10Aa T_0_ GM cotton plants were digested with *Xba*I (unique cutter in the DNA cassette), electrophoresed on 0.8% agarose gel followed by a nylon membrane transference. Blots were probed with a digoxigenin‐labelled 410‐bp Cry10Aa gene (amplified with primers CRY10F and CRY10R; see Table S6) and detected with anti‐digoxigenin antibody conjugated with alkaline phosphatase. WT, wild‐type non‐GM plants.

To determine the copy number of the *cry10Aa* transgene expression cassette in the genome of T_0_ GM cotton, qPCR‐based 2^−ΔΔCt^ was applied. For this, the transgene cassette transcription level was determined by qPCR relative to the expression of a plant endogenous control gene to normalize all reaction variations relative to the initial template DNA concentration differences. Thus, genomic DNA from T_0_ GM cotton plants was used as a template for the qPCR amplification of the *ahas* gene present in the *cry10Aa* transgene cassette and of the plant endogenous *ubc1* gene. The amplification curves for the *ahas* and *ubc1* genes showed good reproducibility. Based on initial template concentration (Log*C*_0_) and linear cycle threshold (*C*_*t*_) values, the following standard curve equations for *ahas* and *ubc1* were obtained: *Y*_AHAS_ = −3.158*x* + 22.29 and *Y*_UBC1_ = −4.2*x* + 27.76, respectively. The coefficients of determination (*R* ^2^) were 0.997 and 0.998 for *ahas* and *ubc1*, respectively, indicating good reproducibility of the linear relationship. Using these equations, the copy number of the *cry10Aa* transgene expression cassette was calculated for the 11 T_0_ GM cotton plants (Figure 4a) and found to be two for 10 of all analyzed GM plants (Figure 4a). The determination of the transgene copy number in T_0_ GM cotton genomes was corroborated by classic Southern blot analysis, which demonstrated that all independent T_0_ cotton transformants presented two *cry10Aa* transgene copies, except for the P#004 event, which presented a single copy of the transgene (Figure 4b).

### Cry10Aa T_0_ GM plants are highly resistant to CBW

**Figure 5:**
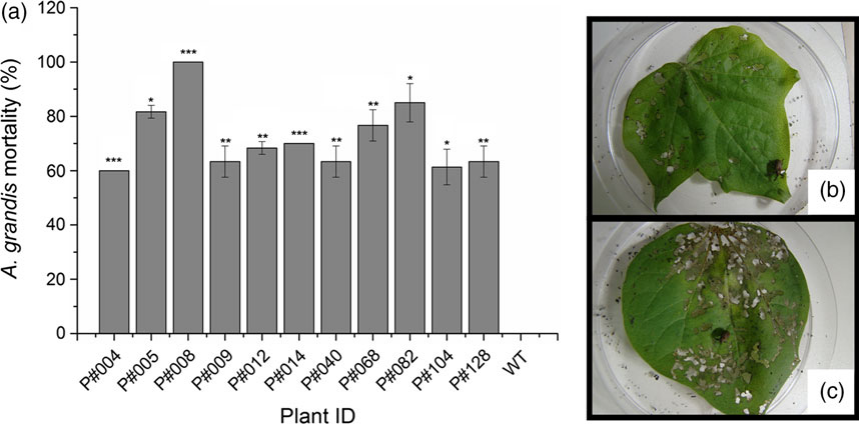
Mortality rate of adult *Anthonomus grandis* fed Cry10Aa T_0_ GM cotton plant leaves. (a) Corrected mortality rate of *A. grandis* fed leaves from eleven Cry10Aa T_0_ GM cotton plants and representative images of the bioassay setup with *A. grandis* fed (b) Cry10Aa GM cotton plant leaves and (c) WT plant leaves. (*) Asterisks represent the level of statistical significance (Student's *t*‐test): (*) *P *≤* *0.05; (**) 0.05 < *P *≤* *0.01; (***) 0.01 < *P *≤ 0.001. WT, wild‐type non‐GM plants.

**Figure 6:**
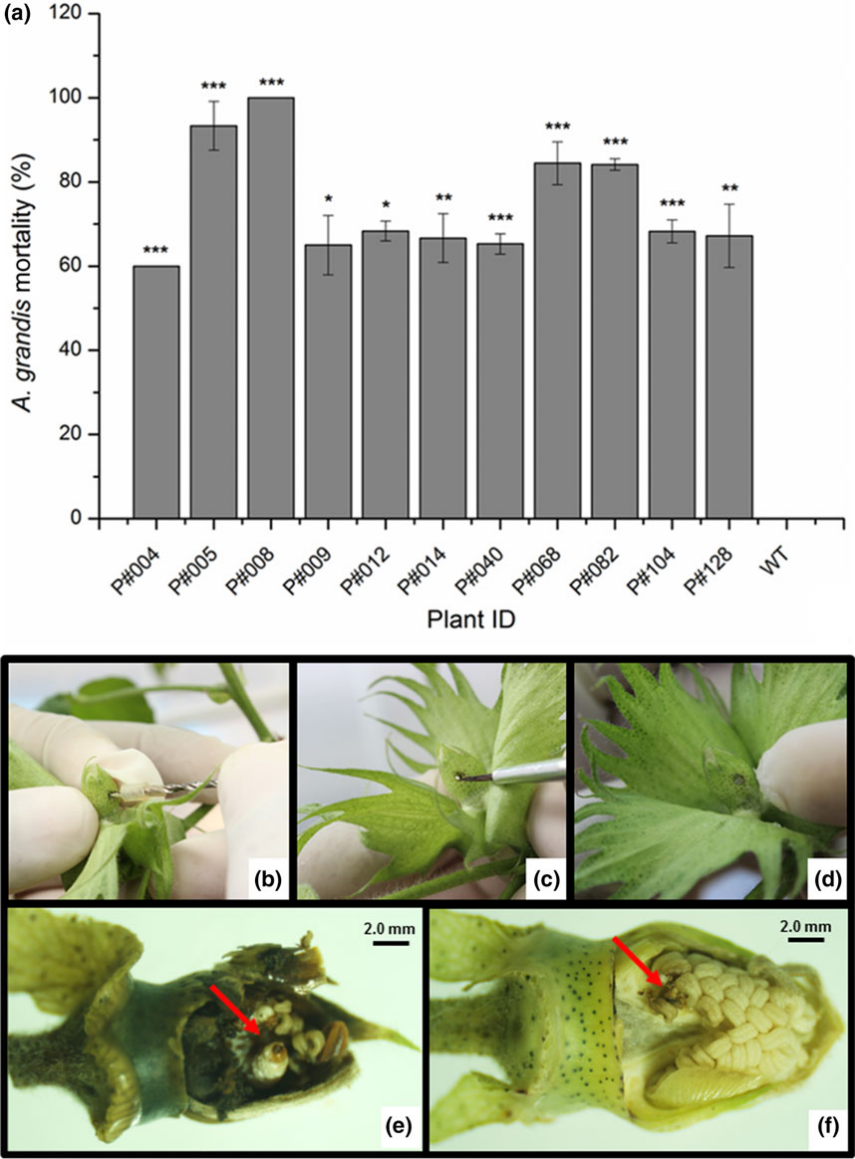
Mortality rate of *Anthonomus grandis* larvae from Cry10Aa T_0_ GM cotton plants. (a) Corrected mortality rate of *A. grandis* fed cotton flower buds from eleven Cry10Aa T_0_ GM cotton plants. One flower bud from each T_0_ plant and WT plant was perforated (b), and one *A. grandis* egg was inoculated (c) and sealed with vaseline (d). Damage into the flower buds collected after egg inoculation from WT and transgenic plants (e and f respectively). The arrows in red indicate larva into the flower buds. (*) Asterisks represent the level of statistical significance (Student's *t*‐test): (*) *P *≤ 0.05; (**) 0.05 < *P *≤ 0.01; (***) 0.01 < *P *≤ 0.001. WT, wild‐type non‐GM plants.

*In planta* bioassays against larvae and adult insects feeding on the leaves (Figures 5, S5) or flower buds (Figures 6, S6) of Cry10Aa T_0_ GM cotton plants were performed to assess the activity of transgenic Cry10Aa toxin against the CBW. The correct mortality (CM) was linearly related to the Cry10Aa effects on adult survival in the leaf‐feeding bioassay and on adult emergence/larval death in the flower bud‐feeding bioassay (Table S3). The mortality rate of adult insects was measured over 15 days during the leaf‐feeding bioassay and ranged from 60% to 100% in GM plants compared to WT plants (Figure 5). A similar trend was observed in larvae under flower bud‐feeding assays, which clearly demonstrates that the Cry10Aa protein is toxic to both CBW larvae and adults, resulting in mortality rates ranging from 60% to 100% within 20 days (Figure 6). There were statistically significant differences between GM and WT plants, highlighting GM P#008 line that produced the highest CBW mortality rate (100%) in both bioassayed tissues. Interestingly, the observed resistance phenotype was correlated with the highest expression levels of the Cry10Aa toxin in the leaves and flower buds of GM P#008 line, which was approximately 14.0 μg g^−1^ fresh tissue in both tissues (Figure 3a, c). Further analysis showed that both damaged leaf area and flower bud drop rates caused by CBW were significantly lower than compared to the same rate in WT plants (Figures S5, S6).

### Analysis of Cry10Aa T_1_ GM plants revealed that CBW resistance is transmitted to the next generation

**Figure 7:**
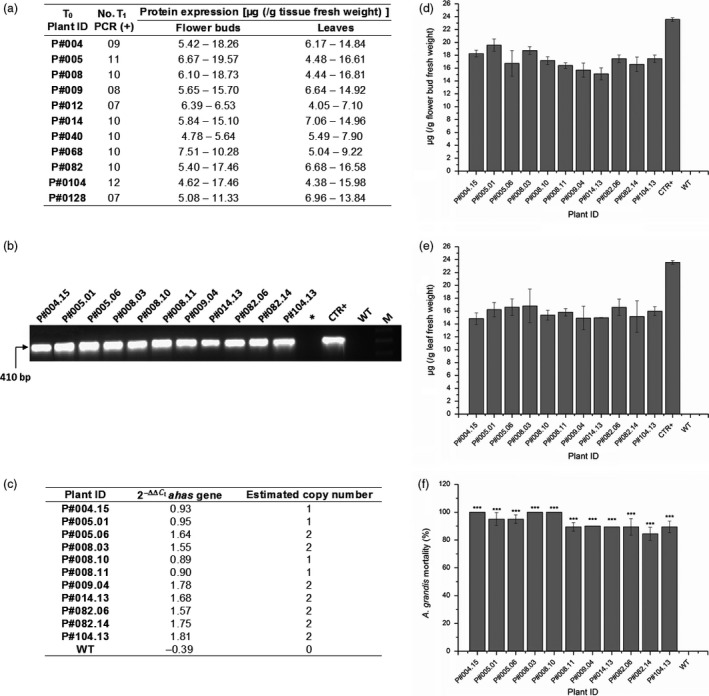
Analysis of Cry10Aa T_1_ GM cotton plants. (a) Experiments summary with Cry10Aa T_1_ GM cotton. Fifteen T_1_ plants were analysed for each respective T_0_ plants, and the number of T_1_ PCR‐positive plants ranged from 7‐12. The Cry10Aa protein expression for each T_1_ PCR‐positive plants was measured by ELISAs both for flower buds and leaves, ranging from 4.05 to 19.57 μg (g tissue fresh weight)^−1^. Among all T_1_ PCR‐positive plants were selected for further analysis those with Cry10Aa protein expression greater than or equal to double of LC_50_ calculated above; (b) detection of positive Cry10Aa T_1_ GM cotton plants via PCR. Genomic DNA was extracted from the leaves of selected plants to amplify the *cry10Aa* gene (410 bp amplicon) in the same conditions that are in Figure 1f. (*) Asterisk represents an empty well; CTR+, positive control (PCR template is the original Cry10Aa expression cassette used for cotton transformation, as presented in Figure 1c; M, 1.0‐kb ladder (INVITROGEN^®^ Cat. # 10787018); (c) copy number of Cry10Aa expression cassette in Cry10Aa T_1_ GM cotton plants by Quantitative PCR (qPCR)‐based 2^−ΔΔCt^ method; (d and e) indirect ELISA quantification of Cry10Aa protein expression in the T_1_ flower buds and leaves respectively. The total protein amount was 1.0 μg per well. CTR+, positive control of recombinant Cry10Aa protein purified of *Escherichia coli*; (f) mortality rate of adult *Anthonomus grandis* fed Cry10Aa T_1_ GM cotton leaves. In all figures, WT means wild‐type non‐GM plants.

To evaluate the stability of *cry10Aa* transgene in subsequent generations, 15 T_1_ plants from each of the eleven T_0_ Cry10Aa GM cotton plants were evaluated (Figure 7). The number of Cry10Aa T_1_ GM plants PCR‐positive for the *cry10Aa* transgene ranged from 7 to 12, which indicate a process of transgene segregation and genotype stabilization (Figure 7a). The Cry10Aa protein expression level in all T_1_ GM plants analysed ranged from 4.62 to 19.57 μg g^−1^ fresh tissue in flower buds and 4.05–16.81 μg g^−1^ fresh tissue in leaves (Figure 7a), which demonstrates a discreet increase in toxin expression levels when compared to T_0_ GM plants (Figure 3).

Among the 165 T_1_ GM plants analysed, 11 were selected to bioassays with CBW (Figure 7b). The selection criteria were based on reduced copy number of transformation cassette in the plant genome (Figure 7c) and toxin expression level in both T_1_ GM flower buds (Figure 7d) and in leaves (Figure 7e) greater than or equal to double of LC_50_ calculated previously in the present work (Table S1).

Regarding bioassays with CBW (Figure 7f; Table S4), three of the eleven plants selected from T_1_ generation (P#004.15, P#008.03 and P#008.10) showed complete resistance to insects (100% CBW mortality rate) and the others (P#005.01, P#005.06, P#008.11, P#009.04, P#014.13, P#082.06, P#082.14 and P#104.13) presented more than 90% CBW mortality rate, which demonstrates the plants ability to transfer for subsequent generation the genotypic/phenotypic CBW resistance trait associated with *cry10Aa* transgene. Furthermore, similar to the analysis with the T_0_ generation, the leaf area damaged by CBW in T_1_ GM cotton leaves was significantly smaller when compared to the damage caused in WT plants (Figure S7).

## Discussion

The Cry10Aa GM plants developed in the present work represent a promising tool for integration into insect pest management (IPM) and cotton breeding programmes due to its high toxicity to CBW. The efficacy of Cry10Aa GM cotton is related to its stable and high protein expression levels driven by the strong and constitutive cotton promoter *uce*A1.7.

The LC_50_ of an *E. coli* recombinant Cry10Aa protein to CBW was similar to the LC_50_ reported by Aguiar *et al*. (2012; Table S1). Moreover, the LC_50_ of the referred *E. coli* recombinant Cry10Aa protein to CBW was lower than that of other Cry toxins described in the literature, which demonstrates the high toxicity of Cry10Aa against *A. grandis*. For instance, Cry1Ia's LC_50_ of 21.5 μg mL^−1^ to CBW neonate larvae and Cry1Ba's LC_50_ of 305.32 μg mL^−1^ to the same insect demonstrate the moderate to no activity of other tested Cry toxins against *A. grandis* (Martins *et al*., 2007, 2010). Thus, the strong toxicity of Cry10Aa makes it a promising molecule for use in the development of GM cotton resistant to CBW. Moreover, genotoxic and hepatotoxic studies of recombinant Cry10Aa revealed that this toxin did not cause mutagenicity in Swiss mice, which highlights the biotechnological potential of Cry10Aa in the development of biosafe GM cotton resistant to CBW (Freire *et al*., 2014).

Mahalakshmi and Shenbagarathai (2010) proposed a structural model for Cry10Aa toxin obtained by homology, for which the template was the Cry2Aa crystal obtained by X‐ray crystallography. In this model, the authors identified the classic three domains present in Cry toxins. As the homology between Cry10Aa and Cry2Aa is low, an alternative predicted structural model based on the Cry1Ac crystal template was presented (Evdokimov *et al*., 2014), which has a higher amino acid sequence identity with Cry10Aa (26.42%; Figure S1a) and is phylogenetically more closely related to Cry1Ac than Cry2Aa (Figure S1d). Although the two models show Cry10Aa with three distinct domains and an unresolved N‐terminus (Figure S2), some differences were observed (Mahalakshmi and Shenbagarathai, 2010): (i) the new model has seven α‐helixes in domain I, while the previous has eight, and (ii) the characteristic C‐terminal α‐helix was better elucidated in the new model (Figure S2b and S2c). Furthermore, (iii) the Cry10Aa domain I had higher structural similarity with the respective domain in Cry2Aa than in Cry1Ac, while domains II and III had greater similarity to Cry1Ac than to Cry2Aa (Figure S2C). These observations could indicate recombination events between related *cry* gene loci in the *Bt* plasmid genome. Thus, the structural elucidation of Cry10Aa using recurrent techniques, such as X‐ray crystallography, is essential for determining the actual toxin structure, which could have implications on the development of GM cotton plants with greater resistance to CBW.

One of the most important factors for achieving the desired expression levels of a transgene is the choice of the promoter (Bruderer *et al*., 2003). *CaMV*35S is a widely used, general‐purpose constitutive promoter in GM plants; however, its stability and expression pattern in heterologous systems present temporal and spatial variation throughout the plant cycle (Kranthi *et al*., 2005; Olsen *et al*., 2005). GM cotton has been approved worldwide (Table S5), and most insecticidal transgenes are driven by *CaMV*35S. There is variability in the toxicity of *Bt* GM cotton against target insects under field conditions (Ma *et al*., 2014). The reduction in Cry toxin biosynthesis in late‐season cotton tissues could be attributed to the overexpression of the *cry* gene in earlier stages, which leads to gene regulation at post‐transcription levels as well as to transgene silencing during later stages (Bakhsh *et al*., 2011; Olsen *et al*., 2005; Ye *et al*., 2009). Puspito *et al*. (2015) reported that 74 GM cotton plants harbouring two genes (*cry1Ac* and *cry2A*) showed decreased toxin levels over time, probably due to either a weak promoter (*CaMV*35S) region or plant ageing. Therefore, the use of stronger and more stable promoters is crucial for reaching high expression levels of the resistance gene in target tissues. In contrast to *CaMV*35S, ubiquitination‐related gene promoters (of ubiquitin‐conjugating enzymes E2) are differentially regulated across tissues and developmental stages (Christensen and Quail, 1996; Mazzucotelli *et al*., 2008). Previous studies identified from *G. hirsutum* the *ghgdrp85* gene, an E2 family member that drives high levels of endogenous expression in various cotton tissues (Artico *et al*., 2013; Viana *et al*., 2011). Reporter gene expression driven by *uce*A1.7 revealed that this cotton promoter is more active in flowers and fruits, the tissues targeted by CBW in cotton (Figure S3). Although some *A. thaliana* tissues have presented reporter gene expression driven by the *CaMV*35S promoter similar to or higher than that driven by the *uce*A1.7 promoter (Figure S3), the choice of the *uce*A1.7 promoter for the Cry10Aa expression cassette used here for cotton transformation is justifiable because *uce*A1.7 is an endogenous promoter, which may prevent future gene silencing in GM progeny. Furthermore, this study demonstrated that the *uce*A1.7 promoter drives high levels of transgene transcription in cotton and these transcripts are translated in functional protein. Therefore, the *uce*A1.7 promoter is a promising regulatory element for the development of CBW‐resistant GM cotton, especially due to the high expression that it drives in cotton reproductive tissues targeted by CBW.

Most commercial cotton cultivars are hardly regenerated into fertile GM plants after *A. tumefaciens*‐mediated transformation. However, stable gene integration and expression have been effectively achieved in different cotton varieties using biolistic method (Aragão *et al*., 2005). The cotton transformation technique associated with Imazapyr herbicide resistance permits high efficient GM plant selection (Aragão *et al*., 2005; Rech *et al*., 2008). The biolistic method could effectively transform the cotton BRS 372 variety with a high transformation rate (0.5%) compared to other techniques, such as the pollen‐tube pathway, which provides very low transformation rates (approximately 0.01%; Oliveira *et al*., 2016).

The qPCR‐based 2^−ΔΔCt^ method for the determination of transgene copy number demonstrated the successful integration of the *uce*A1.7::*cry10*Aa‐*nos*t‐*ahas* cassette into the genome in both T_0_/T_1_ transformed cotton plants (Figure 4, 7c). Despite using a biolistic approach, which usually results in T_0_ plants with more than two transgene copies, T_0_ transformed cotton plants presented either one (a single event) or two (most) copies of the *cry10Aa* transgene (Figure 4). The high reproducibility and sensitivity of qPCR‐based 2^−ΔΔCt^ make this method a reliable alternative for determining the transgene copy number, as Southern blot is unreliable for determining gene copy number under rearrangement or *in tandem* repeats integration (Yang *et al*., 2012).

The Cry1Ac level in Bollgard I GM cotton (Monsanto^®^) is 1.56 and 0.86 μg g^−1^ fresh tissue in leaves and seeds, respectively, and the Cry2Ab2 level in Bollgard II GM cotton (Monsanto^®^) is 8.8 μg g^−1^ pollen fresh weight (Grossi‐de‐Sa *et al*., 2006). Silva *et al*. (2015) and Oliveira *et al*. (2016) reported GM cotton plants with Cry1IA12 transgene levels of 2.7 and 2.26 μg g^−1^ leaf fresh weight, respectively. The highest Cry10Aa expression level observed in the present P#008 GM cotton line (~14.0 μg g^−1^ fresh tissue in both leave and flower buds) clearly shows that Cry10A GM cotton plants can produce toxin at higher levels than usual (Figure 3). This observation is likely partially explained by *uce*A1.7 promoter activity, as other studies reported transgene expression driven by the *CaMV*35S promoter.

The expression profiles of both Cry10Aa transcript and protein were similar (Figures 2, 3). Furthermore, the Cry10Aa transcript and protein expression levels were positively correlated with GM cotton plants conferring resistance to CBW. This observation demonstrates that Cry10Aa expressed in GM cotton triggers CBW mortality.

Even though T_0_ and T_1_ GM cotton plants are heterozygous, analysis of Cry10Aa T_1_ GM generation showed that 100% of the T_0_ GM plants could segregate the *cry10Aa* transgene with a high level of toxin expression. This observation can be related with *uce*A1.7 promoter regulation and by the efficiency of the T_0_ plant selection system via imazapyr associated to the transformation process optimization. According to Aragão *et al*. (2005), this selection system increases about 0.55% the transformation frequency of germ‐lines, which is 100‐fold more efficient when compared to the same system without this selective agent.

At present, there is no commercial cotton cultivar‐either conventional or GM—resistant to CBW. Recent studies have presented Brazilian cotton lines expressing the Cry1Ia12 toxin, which results in up 60% CBW mortality (Oliveira *et al*., 2016; Silva *et al*., 2015). Therefore, the herein presented Cry10Aa GM cotton is promising for further field characterization and eventual commercialization, as these plants reached 100% CBW mortality (Figures 5, 6).

As discussed above, CBW is a major problem for tropical cotton cultivation, whose control is ineffective and costly. Even with the eradication of the CBW in the USA after a robust control programme, the risk of spreading to Africa and Asia is imminent (Azambuja and Degrande, 2014). In this context, the development of the herein presented Cry10Aa GM cotton is promising for the sustainable CBW control.

## Experimental procedures

### Cry10Aa expression cassette for cotton transformation

The Cry10Aa expression cassette for cotton transformation was designed for the expression of the original *cry10Aa* gene sequence of *B. thuringiensis* S1804 strain (Aguiar *et al*., 2012; Thorne *et al*., 1986) under the control of the cotton ubiquitin‐conjugating enzyme promoter *uce*A1.7 (Grossi‐de‐Sa *et al*., 2012), including the nopaline synthase polyadenylation signal terminator (*nos*t) sequence and the mutant acetohydroxyacid synthase (*ahas*) selection marker sequence that confers resistance to imidazolinone herbicides. For *ahas* expression, the corresponding subcassette contained the *ahas* gene promoter, the mutant *A. thaliana ahas* coding sequence, and the *ahas* terminator sequence (Aragão *et al*., 2000). The final expression cassette *uce*A1.7::*cry10Aa‐nos*t‐*ahas* (9024 bp) for cotton transformation was chemically synthesized by Epoch Life Science, Inc. (Texas) and subcloned into the pBluescript SK (−) (pBSK−) vector. The expression cassette was excised from pBSK− by digestion with *BamH*I restriction enzyme prior to cotton transformation.

### Cotton genetic transformation and regeneration

Seeds from the *G. hirsutum* BRS 372 Brazilian variety were manually harvested and delinted with sulphuric acid (3 mL g^−1^ seed) by vigorously stirring for 1 min. The seeds were then immediately transferred to 5 L of distilled water, thoroughly rinsed three times with distilled water and dried in adsorbent paper. Biolistic technique was used to transform *G. hirsutum* with 5 μg of expression cassette *uce*A1.7::*cry10Aa*‐*nos*t‐*ahas* according to Rech *et al*. (2008). In parallel, nontransformed [wild type (WT)] BRS 372 plants were cultivated as negative controls for Cry10Aa GM cotton plants, which were finally acclimated in a greenhouse under similar conditions as mentioned above.

### Selection of Cry10Aa T_0_/T_1_ GM cotton plants

T_0_ GM cotton plants bombarded with the Cry10Aa expression cassette were preselected for tolerance to imidazolinone (presence of a mutant *ahas* gene) by exposing the plants to the commercial herbicide Imazapyr ARSENAL^®^NA (BASF^®^, Germany). After selection of T_0_ cotton plants with promising results in molecular characterization and bioassays, all flower buds that emerged in these plants were sealed to avoid cross‐fertilization. Then, 15 seeds from each selected T_0_ plant were sown and the plantlets were acclimated in greenhouse conditions to represent the T_1_ generation and further characterization.

Genomic DNA from T_0_ and T_1_ GM plants was extracted from newly sprouted leaves from imidazolinone‐tolerant plants or from WT plants using the DNeasy Plant Maxi Kit (QIAGEN^®^, GERMANY) according to the manufacturer's instructions. The genomic DNA from T_0_ GM plants was used to perform PCR to detect the presence of mutant *ahas* and *cry10Aa* genes, while in T_1_ GM plants only the *cry10Aa* gene was analysed. The PCR included a mixture from the Extract‐N‐Amp Plant PCR Kit (SIGMA, St. Louis, Missouri, EUA), 50 ng of genomic DNA from putative GM or WT plants and the primers listed in Table S6 (CRY10F, CRY10R, AHASF and AHASR). The PCR conditions were as follows: (i) to detect the mutant *cry10Aa* gene, 95 °C for 5 min, 40 cycles (94 °C for 45 s, 57 °C for 45 s and 72 °C for 1 min), and 72 °C for 10 min for final extension; and (ii) to detect the mutant *ahas* gene, 95 °C for 5 min, 32 cycles (95 °C for 1 min, 57 °C for 30 s and 72 °C for 1 min), and 72 °C for 10 min for final extension. Genomic DNA from WT plants was used as a negative control, while the original Cry10Aa expression cassette used for cotton transformation was used as a positive control. The PCR products were analysed by agarose gel electrophoresis.

### Quantitative PCR (qPCR) assays

Total RNA of each foliar replicate (i.e. a foliar disc of 1 cm in diameter) of Cry10Aa T_0_ GM cotton plants was isolated using Concert (INVITROGEN, Carlsbad, Califórnia, EUA^®^) following the manufacturer's instructions. All of the RNA extractions were performed in biological triplicate. Prior to the cDNA synthesis, all of the RNA samples were treated with 1 U of Ambion^®^ DNase I RNase‐free™ (INVITROGEN^®^). The cDNA was retro transcribed from 1.0 μg of total RNA using 200 U of *Moloney Murine Leukaemia Virus Reverse Transcriptase* (M‐MLV RT) (INVITROGEN^®^) and oligo‐dTNVd_30_, following the instructions of the M‐MLV RT manufacturer. The primers used in these experiments are presented in Table S6 The fluorescence raw data from all qPCR amplification runs were imported into the Real‐time PCR Miner software (Zhao and Fernald, 2005) to determine the *C*_*t*_ value and qPCR efficiency. Gene expression analyses were completed using qBASE Plus software (Hellemans *et al*., 2007). Statistical analysis was performed using the REST software (QIAGEN^®^, Germany).

### Immunodetection of the Cry10Aa protein from the T_0_/T_1_ GM cotton leaves and flower buds

The anti‐Cry10Aa polyclonal antibody (pabCry10Aa) was produced in rabbit by GenScript^©^ (New Jersey) from a synthetic peptide (VSSDSKIVKGPGHT) based on the Cry10Aa amino acid sequence and *in silico* immunogenic studies.

For the Western blot immunodetection of Cry10Aa in T_0_ GM plants, total protein extracts were obtained either from fresh leaves (3 g) or flower buds (0.6 mg) grounded in liquid nitrogen. These materials were homogenized in 15 mL of total protein extraction buffer [Tris‐buffered saline (TBS) (0.5 m Tris‐OH, 1.5 m NaCl, pH 7.5) added to 0.5% (w/v) ascorbic acid, 10 mm sodium metabisulphite, 0.5% (v/v) Triton X‐100 and 1 mm PMSF]. After 2 h under slow stirring at 4 °C, the homogenate was centrifuged twice at 5000× ***g*** for 10 min at 4 °C. The proteins in the total protein extracts were quantified using Bradford's method (Bradford, 1976) based on a bovine serum albumin (BSA) standard curve. For the Western blot immunodetection of Cry10Aa, 20.0 μg of each total protein extract was subjected to 10.0% SDS‐PAGE (Laemmli, 1970). After electrophoresis, the separated proteins were transferred from the gel to a nitrocellulose membrane (HYBOND^®^, GE HEALTHCARE^®^, UK) using semi‐dry TransBlot Cell Unit (BIO‐RAD^®^) (Towbin *et al*., 1979). The membrane was blocked overnight in 3.0% (w/v) gelatine in TBS added to 0.05% (v/v) Tween‐20 and 0.5% (w/v) polyvinyl alcohol (PVA) at room temperature. After washing three times in TBS–Tween‐20 and once in TBS, the membrane was incubated in 2.0% (w/v) gelatine in TBS with pabCry10Aa primary antibody (1 : 1000) for 2 h at room temperature. Subsequently, rapid washing was performed as mentioned above, and the membrane was incubated in anti‐rabbit IgG alkaline phosphatase‐conjugated (BIO‐RAD^®^) secondary antibody (1 : 3000) for 1 h at room temperature. Finally, the Alkaline Phosphatase Detection kit (BIO‐RAD^®^) was used for the colorimetric detection of Cry10Aa according to the manufacturer’ instructions.

For ELISA immunodetection and quantification of Cry10Aa in T_0_/T_1_ GM cotton plants, fresh leaf and flower bud (0.6 mg) materials were transferred to microtubes and homogenized in 1 mL of total protein extraction buffer [0.7 m sucrose, 0.5 m Tris, 50 mm EDTA, 0.1 m KCl, 0.5% (v/v) Triton X‐100, 1 mm DTT, 1 mm PMSF, pH 8.0] with 0.05% (w/v) polyvinylpyrrolidone (PVPP) added just before use. The total protein extracts were then stirred for 20 min at 4 °C. The supernatants were collected after centrifugation at 9000× ***g*** for 4 h at 4 °C. Entire 96‐wells microtiter plate was coated with 1.0 μg of total protein extracts from fresh flower buds and leaves, either Cry10Aa GM cotton or WT plants. The microplates were incubated for 1 h at 37 °C and, subsequently, nonspecific binding sites were blocked by incubation in 3.0% BSA dissolved in PBS (100 mm NA_2_HPO_4_, 17 mm KH_2_PO_4_, 5 m NaCl, 27 mm KCl, pH 7.4) added to 0.05% Tween‐20 overnight at 37 °C. Next, microplate wells were incubated with pabCry10Aa primary antibody (1 : 1000 in 2.0% BSA‐PBS) for 2 h at 37 °C. After washing (PBS added to 0.05% Tween‐20), the microplate wells were incubated with anti‐rabbit IgG horseradish peroxidase (HRP)‐conjugated (BIO‐RAD^®^) secondary antibody (1 : 3000 in 1.0% BSA‐PBS added to 0.05% Tween‐20) for 2 h at 37 °C. After washing as described above, Cry10Aa was detected in colorimetric assays by incubating the microplate wells in tetramethylbenzidine dihydrochloride (TMD) [one tablet of TMD dissolved in 10 mL of buffer detection buffer (0.2 m Dibasic Sodium Phosphate, 0.1 m Citric Acid, pH 5.0)]. The colorimetric detection reaction was stopped using 3 m H_2_SO_4_. ELISA was carried out in biological and technical triplicate. The absorbance of the developed colour was measured at 450 nm. Recombinant Cry10Aa produced in *E. coli* was used to obtain a standard curve ranging from 0 to 300 μg Cry10Aa. All the data were statistically analysed by ANOVA software, and the means were compared by Student's *t*‐test with 0.05 probability, with the control sample having the lowest expression level (excluding WT samples). The classification relating the Cry10Aa toxin production levels with the number of transgene copies in transgenic GM cotton genomes (shown in the graphs presenting the T_0_ ELISA results) was obtained based on statistical analysis with Tukey's test. In these analyses, the lowest expression level (excluding WT samples) was compared with all others.

### Determination of Cry10Aa transgene expression cassette copy number in the genome of T_0_/T_1_ GM cotton plants by qPCR‐based 2^−ΔΔCt^

The copy number of the Cry10Aa transgene expression cassette in the genome of T_0_/T_1_ GM cotton plants was determined according to Yang *et al*. (2012) using qPCR‐based 2^−ΔΔCt^ method. For this purpose, genomic DNA from WT and Cry10Aa GM cotton plants was extracted from fresh leaves (100 mg) using the DNeasy Plant Maxi Kit (QIAGEN^®^, GERMANY) according to the manufacturer's instructions. The DNA concentration was determined spectrophotometrically, and the DNA integrity was checked by agarose gel electrophoresis. A reference plasmid denoted pBSK‐*ahas*‐*ubc1* containing a fragment of the *G. hirsutum* ubiquitin C gene (*ubc1*), which is a single‐copy gene, and the selection marker gene *ahas*, which was also present in the Cry10Aa expression cassette for cotton transformation, was constructed. The relative qPCR quantification of *ahas* against the endogenous reference gene *ubc1* was used to calculate the copy number of the Cry10Aa transgene expression cassette in the genome of T_0_/T_1_ GM cotton plants using the primers AHASF, AHASR, GhUBC1F and GhUBC1R (Table S6). First, the absolute quantity of the two genes *ubc1* and *ahas* was determined by qPCR in reference to standard curves. Two standard curves were obtained by plotting the threshold cycle or *C*_*t*_ values against the log‐transformed concentration of serial tenfold dilutions (10^1^, 10^2^, 10^3^, 10^4^ and 10^5^) from the same reference plasmid, pBSK‐*ahas*‐*ubc1*. The absolute copy number of the plasmid pBSK‐*ahas*‐*ubc1* was calculated using *C*_*t*_ values based on standard curves. The relative copy number of the *ahas* target gene was obtained by dividing the absolute concentration of the *ahas* target gene by that of the *ubc1* endogenous gene in the same sample.

### Determination of Cry10Aa transgene expression cassette copy number in the genome of T_0_ GM cotton transformants by Southern blot

For genomic Southern blot (Sambrook and Russell, 2001), total DNA (20 μg) was digested with *Xba*I (unique cutter in the DNA cassette), electrophoresed on 0.8% agarose gel followed by a nylon membrane transference. Blots were probed with a digoxigenin‐labelled 410‐bp Cry10Aa gene (amplified with primers CRY10F and CRY10R; see Table S6). The hybridized digoxigenin‐labelled probes were detected with anti‐digoxigenin antibody conjugated with alkaline phosphatase (AP) and revealed using an AP conjugate substrate kit (BIO‐RAD^®^).

### *In planta* bioassays against CBW larvae and adult insects

Both Cry10Aa GM and WT cotton plants were subjected to two different types of bioassays against CBW: a bioassay in flower buds against CBW larvae and a bioassay in leaves against adult insects. For these assays, a population of *A. grandis* was maintained at the insect rearing platform at Embrapa Genetic Resources and Biotechnology (Brasília, Brazil) on a standard rearing diet at 27 ± 2 °C, 70 ± 10% relative humidity, and a photoperiod of 14 h of light (Monnerat *et al*., 2000).

For the bioassay in flower buds against CBW larvae, the reproductive structure with approximately 6 mm in diameter from either Cry10Aa T_0_ GM or WT cotton plants cultivated under greenhouse conditions were perforated with a drill just above the floral receptacle. Subsequently, a CBW egg containing an active CBW embryo was introduced into the drilled orifice, and the region was sealed with vaseline to prevent egg dehydration before larva eclosion. After 20 days, the mortality rate of larvae and the flower bud drop rate were measured. The experiment was carried out in triplicate. Each experimental unit consisted of ten flower buds per plant.

For the bioassay in leaves against CBW adults, adult insects were fed with detached leaves from either Cry10Aa T_0_/T_1_ GM or WT cotton plants cultivated under greenhouse conditions. One adult insect was released per Petri dish containing a single young leaf, and the plates were incubated in an acclimatized room at 27 ± 2 °C with photoperiod of 14 h of light. The leaf of each bioassay was replaced by a fresh one every 3 days. Observations of the CBW behaviour, morphology, mortality and other parameters were recorded daily. After 15 days, the CBW adult mortality rate was calculated. This bioassay was performed in triplicate, and each experimental unit consisted of 100 Petri dishes. All the leaves collected during the bioassays were scanned and the percentage of damaged area by the insects in each T_0_/T_1_ GM or WT cotton plants was measured using ImageJ software (Schneider *et al*., 2012), through the formula

$$\frac{\mathrm{damaged}\;\mathrm{leaf}\;\mathrm{area}}{\mathrm{total}\;\mathrm{leaf}\;\mathrm{area}}\times 100.$$

All data were statistically analysed by ANOVA, and the means were compared by Student's *t*‐test with 0.05 probability. The CM rate of the CBW was estimated using Schneider–Orelli's formula (Schneider‐Orelli, 1947):

CM(%)=(T−C)/(100−C)×100,

where *T* (%) is the CBW mortality rate in Cry10Aa T_0_/T_1_ GM plants (bioassay treatment) and *C* (%) is the CBW mortality rate in WT cotton plants (bioassay negative control).

## Conflict of interests

The authors declare no conflict of interests.

## Supporting information


**Figure S1** Template determination for drafting the Cry10Aa *in silico* three‐dimensional model. The Cry10Aa sequence (Thorne *et al*., 1986; *Journal of Bacteriology* **166**, 801‐811; Aguiar *et al*., 2012, *Bt Research* **3**, 20‐28) and the crystal structure of Cry1Ac (PDB ID: 4W8J; solved at 2.78 Å) were used to generate the Cry10Aa three‐dimensional structure model. To choose the best template, that is either Cry1Ac or Cry2Aa (PDB ID: 1I5P), the protein sequences of candidate templates were aligned using MUSCLE software (Edgar, 2004, *Nucleic Acids Research* **32**, 1792‐1797), and the phylogenetic relationship between them was determined using MEGA 6 software (Tamura *et al*., 2013, *Molecular Biology and Evolution* **30**, 2725–2729) using the neighbour‐joining and bootstrap phylogenetic methods with 1000 replications. Structural elucidation was performed using a homology modelling approach with Modeller 9v8 (Sali, 1995, *Current Opinion in Biotechnology* **6**, 437‐451) and Swiss‐Model (Biasini *et al*., 2014, *Nucleic Acids Research* **42**, W252–W258). Cry10Aa secondary structure was predicted using the PDBsum software (Laskowski, 2007, *Bioinformatics* **23**, 1824‐1827). **(a)** Multiple alignment of the amino acid sequences of the two template candidates (Cry1Ac and Cry2Aa) with the Cry10Aa toxin. The alignment coverage values of Cry10Aa with Cry1Ac and Cry2Aa were 0.86 (22‐678) and 0.38 (27‐321), respectively. The identity values were 26.42% and 17.31%, respectively. **(b)** Predicted tertiary structure of Cry10Aa (accession number AAA22614.1) based on the Cry1Ac crystal (PDB ID: 4W8J), presenting the three typical Cry domains I, II and III. The depicted C‐terminal α‐helix indicates Cry pro‐toxin. The model shows a Cry typical 3D‐deltaendotoxin (three‐domain) conformation, typical of pore‐forming toxins, with seven helixes in domain I, three beta sheets in domain II and a beta sandwich in domain III. The Cry10Aa model presents an extra C‐terminal α‐helix and an N‐terminal loop, both typical of Cry pro‐toxins; **(c)** superposition of the two models of Cry10Aa toxin obtained with Cry1Ac (in brown) and Cry2Aa (in blue) as templates. In the Cry1Ac‐based Cry10Aa model, the C‐terminal α‐helix was better resolved than in the Cry2Aa‐based Cry10Aa model. Furthermore, the Cry10Aa domain I has higher structural similarity with the respective domain in Cry2Aa, while domains II and III have greater similarity to the cognate regions of the Cry1Ac protein; **(d)** phylogenetic relationship among Cry1Ac, Cry2Aa and Cry10Aa toxins (outgroup—Cyt‐like protein—accession number AAB49768.1). The bar indicates the phylogenetic distance between the sequences.

**Figure S2** Prediction of Cry10Aa toxin secondary structure. Representation of Cry10Aa (accession number AAA22614.1) secondary structure obtained from *in silico* modelling using Cry1Ac crystal (PDB ID: 4W8J) as a template. The 66 first residues present in the Cry10Aa protein do not show structural similarity with Cry1Ac crystal. Predicted helixes are represented as coils. Predicted sheets are represented as arrows. Helixes are labelled H1 to H19. Sheets are labelled A to E. The motifs are indicated as follows: *β*: beta turn; *γ*: gamma turn; and *hairpin*: beta hairpin. Analyses were performed using PDBsum (https://www.ebi.ac.uk/pdbsum/).

**Figure S3** Analyses of candidate promoters supporting the design of the Cry10Aa expression cassette for cotton transformation. The cotton ubiquitin‐conjugating enzyme (*uce*A) constitutive promoter, rather than *CaMV*35S, was used to design the transformation vector. This choice was based on comparative results of the strength and tissue specificity of the *CaMV*35S and *uce*A1.7 promoters, and the *uid*A reporter gene (i.e. GUS‐encoding gene) expression levels driven by both promoters was evaluated in GM *A. thaliana*. For this, *uidA* mRNA levels in T_3_ GM *A. thaliana* plants driven by either *CaMV*35S or *uce*A1.7 were measured in several tissues (flower buds, inflorescences, open flowers, siliques/fruits, roots, stems and leaves) through qPCR. Although the *uidA* mRNA levels driven by the cotton *uce*A1.7 promoter were similar to those driven by the *CaMV*35S promoter in all flower stages, the *uce*A1.7 promoter could drive higher levels of *uidA* transcripts than could the *CaMV*35S promoter in fruits (sevenfold) and vegetative tissues, such as the roots and stem (twofold). These results suggest that the *uce*A1.7 promoter is probably stronger and more specific than *CaMV*35S in cotton fruits, which is the main tissue target of the CBW. As the aim of this study is cotton protection against CBW, Cry10Aa expression driven by the *uce*A1.7 promoter is probably more appropriate than that driven by the *CaMV*35S promoter in GM cotton. Therefore, the cotton *uce*A1.7 promoter, rather than *CaMV*35S, was used to design the Cry10Aa transformation cassette. The qPCR experimental procedures were as described for Cry10Aa GM cotton plants. The primers used in these experiments are presented in Table S6 The asterisks represent the level of statistical significance between *uid*A expression driven by either the *CaMV*35S or *uce*A1.7 promoter (Student's *t*‐test): (*) *p *≤* *0.05; (**) 0.05 <  *p *≤* *0.01; (***) 0.01 <  *p *≤* *0.001. After normalization based on the expression of plant endogenous genes, the values were plotted relative to the lowest expression value (excluding the WT non‐transformed plants) set as expression level 1 (one).

**Figure S4** Phenotypic comparison between T_0_ Cry10Aa cotton plants and nontransgenic plants. The images compare the macroscopic phenotypes of Cry10Aa cotton P#008 with wild type (WT—BRS 372), both at 4 months of age. In (**a**) is shown the view of the whole aerial part of transgenic (T) and non‐trangenic (WT) plants. In (**b**) and (**c**), the floral structures of WT and P#008 respectively are compared, whereas in (**d**) and (**e**) the leaves of the same plants are compared. Both T and WT plants have a medium‐late life cycle, and all vegetative (aerial) and reproductive structures are morphologically similar. This comparison extends to the other T_0_ plants (P#004, P#005, P#009, P#012, P#014, P#040, P#068, P#082, P#104 and P#128).

**Figure S5** Leaf area damaged by CBW in T_0_ GM cotton plants. The graph shows the percentage of leaf area damaged by CBW in leaves of eleven T_0_ GM cotton plants expressing the toxin Cry10Aa and WT (control), all collected every three days during the bioassays. The rate (damaged leaf area/total leaf area) was obtained using ImageJ software (Schneider *et al*., 2012). (*) Asterisks represent the level of statistical significance when compared to the damage in WT plants (Student's *t*‐test): (*) *p *≤* *0.05; (**) 0.05 <  *p *≤* *0.01; (***) 0.01 <  *p *≤* *0.001. **Abbreviation: WT**—wild‐type non‐GM plants.

**Figure S6** Flower bud drop rate in T_0_ GM cotton plants infested with CBW. The graph shows the percentage of dropped flower buds in eleven T_0_ GM cotton plants expressing the toxin Cry10Aa and WT (control) evaluated during the bioassays. (*) Asterisks represent the level of statistical significance when compared to the same rate in WT plants (Student's *t*‐test): (*) *p *≤* *0.05; (**) 0.05 <  *p *≤* *0.01; (***) 0.01 <  *p *≤* *0.001. **Abbreviation: WT**—wild‐type non‐GM plants.

**Figure S7** Leaf area damaged by CBW in T_1_ GM cotton plants. The graph shows the percentage of leaf area damaged by CBW in leaves of eleven T_1_ GM cotton plants expressing the toxin Cry10Aa and WT (control), all collected every three days during the bioassays. The rate (damaged leaf area/total leaf area) was obtained using ImageJ software (Schneider *et al*., 2012). (*) Asterisks represent the level of statistical significance when compared to the damage in WT plants (Student's *t*‐test): (*) *p *≤* *0.05; (**) 0.05 <  *p *≤* *0.01; (***) 0.01 <  *p *≤* *0.001. **Abbreviation: WT**—wild‐type non‐GM plants.

**Table S1** LC_50_ values of recombinant Cry10Aa protein against *Anthonomus grandis*

**Table S2** Summary of qPCR experiments—*cry10Aa* transcript quantification.

**Table S3** Mortality rate (%) of cotton boll weevil adults fed tissues from T_0_ GM and non‐GM cotton plants.

**Table S4** Mortality rate (%) of cotton boll weevil adults fed tissues from T_1_ GM and non‐GM cotton plants.

**Table S5** Commercially approved *Bt* cotton plants worldwide.

**Table S6** Summary of used primers.
