# Supplementary material for: Transgenic cotton expressing Cry10Aa toxin confers high resistance to the cotton boll weevil
Source: Plant Biotechnol J. 2017 Mar 2;15(8):997–1009. doi: 10.1111/pbi.12694 (PMC5506659; doi:10.1111/pbi.12694)
Supplement: Supplementary file 1 — Figure S1 Template determination for drafting the Cry10Aa in silico three‐dimensional model. The Cry10Aa sequence (Thorne et al., 1986; Journal of Bacteriology 166, 801‐811; Aguiar et al., 2012, Bt Research 3, 20‐28) and the crystal structure of Cry1Ac (PDB ID: 4W8J; solved at 2.78 Å) were used to generate the Cry10Aa three‐dimensional structure model. To choose the best template, that is either Cry1Ac or Cry2Aa (PDB ID: 1I5P), the protein sequences of candidate templates were aligned using MUSCLE software (Edgar, 2004, Nucleic Acids Research 32, 1792‐1797), and the phylogenetic relationship between them was determined using MEGA 6 software (Tamura et al., 2013, Molecular Biology and Evolution 30, 2725–2729) using the neighbour‐joining and bootstrap phylogenetic methods with 1000 replications. Structural elucidation was performed using a homology modelling approach with Modeller 9v8 (Sali, 1995, Current Opinion in Biotechnology 6, 437‐451) and Swiss‐Model (Biasini et al., 2014, Nucleic Acids Research 42, W252–W258). Cry10Aa secondary structure was predicted using the PDBsum software (Laskowski, 2007, Bioinformatics 23, 1824‐1827). (a) Multiple alignment of the amino acid sequences of the two template candidates (Cry1Ac and Cry2Aa) with the Cry10Aa toxin. The alignment coverage values of Cry10Aa with Cry1Ac and Cry2Aa were 0.86 (22‐678) and 0.38 (27‐321), respectively. The identity values were 26.42% and 17.31%, respectively. (b) Predicted tertiary structure of Cry10Aa (accession number AAA22614.1) based on the Cry1Ac crystal (PDB ID: 4W8J), presenting the three typical Cry domains I, II and III. The depicted C‐terminal α‐helix indicates Cry pro‐toxin. The model shows a Cry typical 3D‐deltaendotoxin (three‐domain) conformation, typical of pore‐forming toxins, with seven helixes in domain I, three beta sheets in domain II and a beta sandwich in domain III. The Cry10Aa model presents an extra C‐terminal α‐helix and an N‐terminal loop, both typical of Cry pro‐toxins; (c [file PBI-15-997-s006.docx]

| 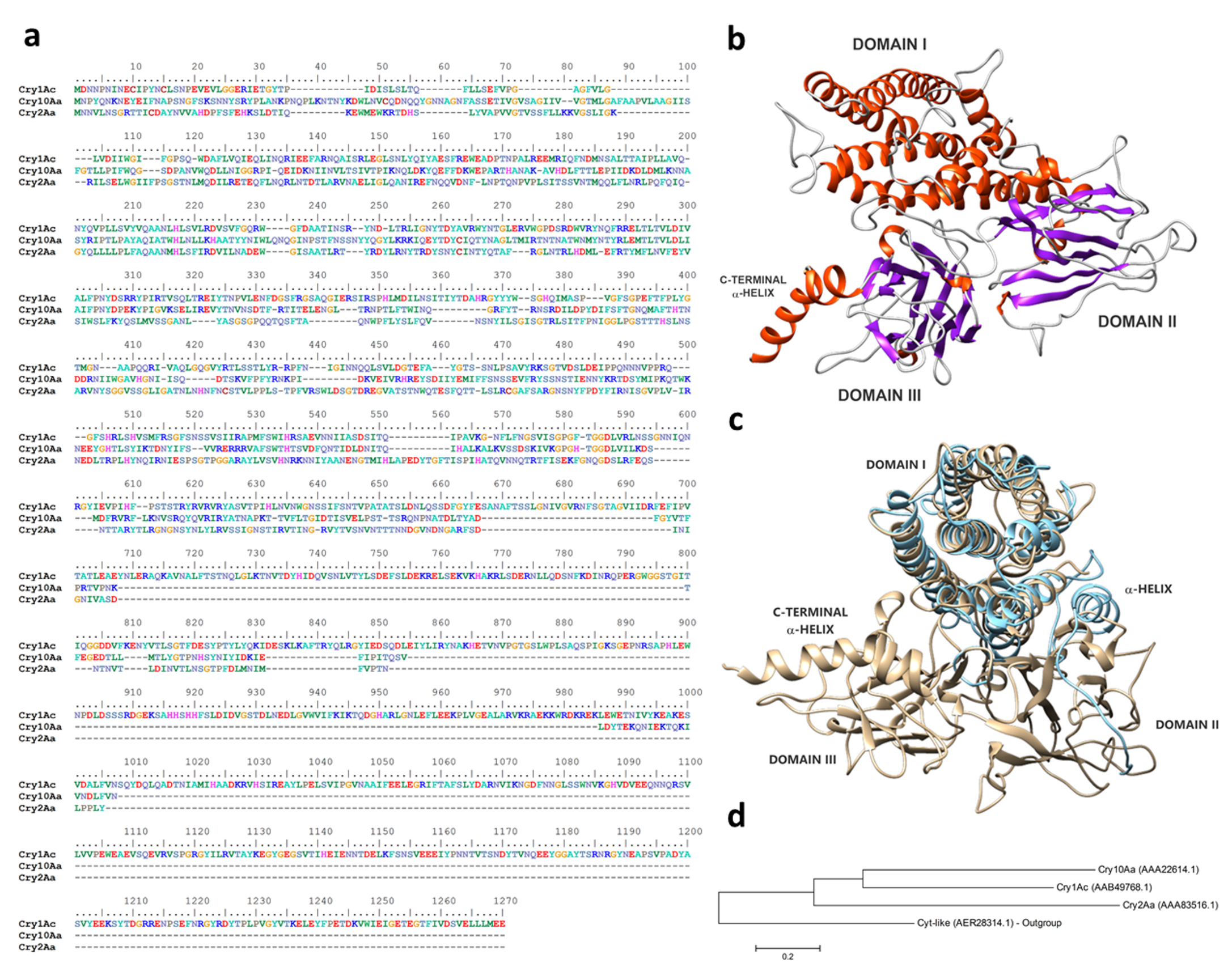  **Figure S1. Template determination for drafting the Cry10Aa *in silico* three-dimensional model**. The Cry10Aa sequence (Thorne et al., 1986, *Journal of Bacteriology* **166**, 801-811; Aguiar et al., 2012, *Bt Research* **3**, 20-28) and the crystal structure of Cry1Ac (PDB ID: 4W8J; solved at 2.78 Å) were used to generate the Cry10Aa three-dimensional structure model. In order to choose the best template, i.e., either Cry1Ac or Cry2Aa (PDB ID: 1I5P), the protein sequences of candidate templates were aligned using MUSCLE software (Edgar, 2004, *Nucleic Acids Research* **32**, 1792-1797), and the phylogenetic relationship between them was determined using MEGA 6 software (Tamura et al., 2013, *Molecular Biology and Evolution* **30**, 2725–2729) using the Neighbour-joining and Bootstrap phylogenetic methods with 1000 replications. Structural elucidation was performed using a homology modelling approach with Modeller 9v8 (Sali, 1995, *Current Opinion in Biotechnology* **6**, 437-451) and Swiss-Model (Biasini et al., 2014, *Nucleic Acids Research* **42**, W252–W258). Cry10Aa secondary structure was predicted using the PDBsum software (Laskowski, 2007, *Bioinformatics* **23**, 1824-1827). **(a)** Multiple alignment of the amino acid sequences of the two template candidates (Cry1Ac and Cry2Aa) with the Cry10Aa toxin. The alignment coverage values of Cry10Aa with Cry1Ac and Cry2Aa were 0.86 (22-678) and 0.38 (27-321), respectively. The identity values were 26.42% and 17.31%, respectively. **(b)** Predicted tertiary structure of Cry10Aa (accession number AAA22614.1) based on the Cry1Ac crystal (PDB ID: 4W8J), presenting the three typical Cry domains I, II and III. The depicted C-terminal α-helix indicates Cry pro-toxin. Cry10Aa amino acid sequence and secondary structure are presented in Figure S4. The model shows a Cry typical 3D-deltaendotoxin (three-domain) conformation, typical of pore-forming toxins, with seven helixes in domain I, three beta sheets in domain II and a beta sandwich in domain III. The Cry10Aa model presents an extra C-terminal α-helix and an N-terminal loop, both typical of Cry pro-toxins; **(c)** Superposition of the two models of Cry10Aa toxin obtained with Cry1Ac (in brown) and Cry2Aa (in blue) as templates. In the Cry1Ac-based Cry10Aa model, the C-terminal α-helix was better resolved than in the Cry2Aa-based Cry10Aa model. Furthermore, the Cry10Aa domain I has higher structural similarity with the respective domain in Cry2Aa, while domains II and III have greater similarity to the cognate regions of the Cry1Ac protein; **(d)** Phylogenetic relationship among Cry1Ac, Cry2Aa and Cry10Aa toxins (outgroup - Cyt-like protein - accession number AAB49768.1). The bar indicates the phylogenetic distance between the sequences. |
| --- |
