# Supplementary material for: Transgenic cotton expressing Cry10Aa toxin confers high resistance to the cotton boll weevil
Source: Plant Biotechnol J. 2017 Mar 2;15(8):997–1009. doi: 10.1111/pbi.12694 (PMC5506659; doi:10.1111/pbi.12694)
Supplement: Supplementary file 2 — Figure S2 Prediction of Cry10Aa toxin secondary structure. Representation of Cry10Aa (accession number AAA22614.1) secondary structure obtained from in silico modelling using Cry1Ac crystal (PDB ID: 4W8J) as a template. The 66 first residues present in the Cry10Aa protein do not show structural similarity with Cry1Ac crystal. Predicted helixes are represented as coils. Predicted sheets are represented as arrows. Helixes are labelled H1 to H19. Sheets are labelled A to E. The motifs are indicated as follows: β: beta turn; γ: gamma turn; and hairpin: beta hairpin. Analyses were performed using PDBsum (https://www.ebi.ac.uk/pdbsum/). [file PBI-15-997-s011.docx]

| 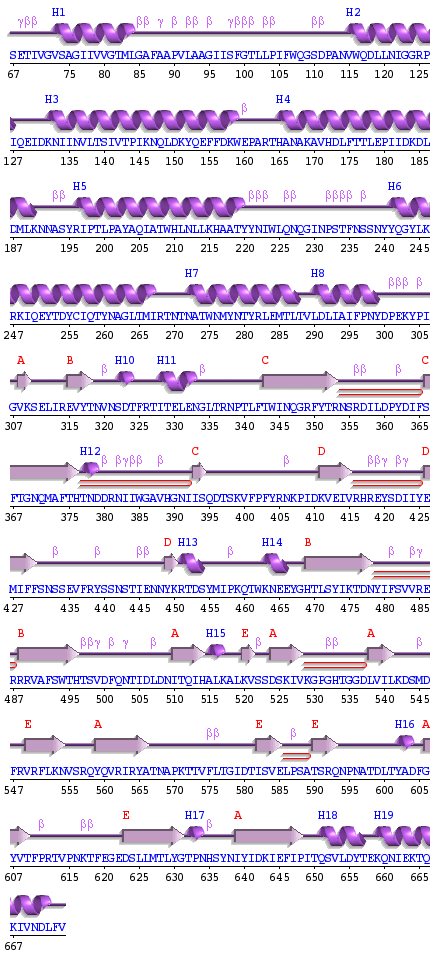   \| **Figure S2. Prediction of Cry10Aa toxin secondary structure.** Representation of Cry10Aa (accession number AAA22614.1) secondary structure obtained from *in silico* modelling using Cry1Ac crystal (PDB ID: 4W8J) as a template. The 66 first residues present in the Cry10Aa protein do not show structural similarity with Cry1Ac crystal. Predicted helixes are represented as coils. Predicted sheets are represented as arrows. Helixes are labelled H1 to H19. Sheets are labelled A to E. The motifs are indicated as follows: *β:* beta turn; *γ:* gamma turn; and *hairpin:* beta hairpin. Analyses were performed using PDBsum (https://www.ebi.ac.uk/pdbsum/). \| \| --- \| |
| --- | --- |
