# Supplementary material for: Transgenic cotton expressing Cry10Aa toxin confers high resistance to the cotton boll weevil
Source: Plant Biotechnol J. 2017 Mar 2;15(8):997–1009. doi: 10.1111/pbi.12694 (PMC5506659; doi:10.1111/pbi.12694)
Supplement: Supplementary file 3 — Figure S3 Analyses of candidate promoters supporting the design of the Cry10Aa expression cassette for cotton transformation. The cotton ubiquitin‐conjugating enzyme (uceA) constitutive promoter, rather than CaMV35S, was used to design the transformation vector. This choice was based on comparative results of the strength and tissue specificity of the CaMV35S and uceA1.7 promoters, and the uidA reporter gene (i.e. GUS‐encoding gene) expression levels driven by both promoters was evaluated in GM A. thaliana. For this, uidA mRNA levels in T3 GM A. thaliana plants driven by either CaMV35S or uceA1.7 were measured in several tissues (flower buds, inflorescences, open flowers, siliques/fruits, roots, stems and leaves) through qPCR. Although the uidA mRNA levels driven by the cotton uceA1.7 promoter were similar to those driven by the CaMV35S promoter in all flower stages, the uceA1.7 promoter could drive higher levels of uidA transcripts than could the CaMV35S promoter in fruits (sevenfold) and vegetative tissues, such as the roots and stem (twofold). These results suggest that the uceA1.7 promoter is probably stronger and more specific than CaMV35S in cotton fruits, which is the main tissue target of the CBW. As the aim of this study is cotton protection against CBW, Cry10Aa expression driven by the uceA1.7 promoter is probably more appropriate than that driven by the CaMV35S promoter in GM cotton. Therefore, the cotton uceA1.7 promoter, rather than CaMV35S, was used to design the Cry10Aa transformation cassette. The qPCR experimental procedures were as described for Cry10Aa GM cotton plants. The primers used in these experiments are presented in Table S6 The asterisks represent the level of statistical significance between uidA expression driven by either the CaMV35S or uceA1.7 promoter (Student's t‐test): (*) p ≤ 0.05; (**) 0.05 < p ≤ 0.01; (***) 0.01 < p ≤ 0.001. After normalization based on the expression of plant endogenous genes, the values were plotted relative t [file PBI-15-997-s010.docx]

| 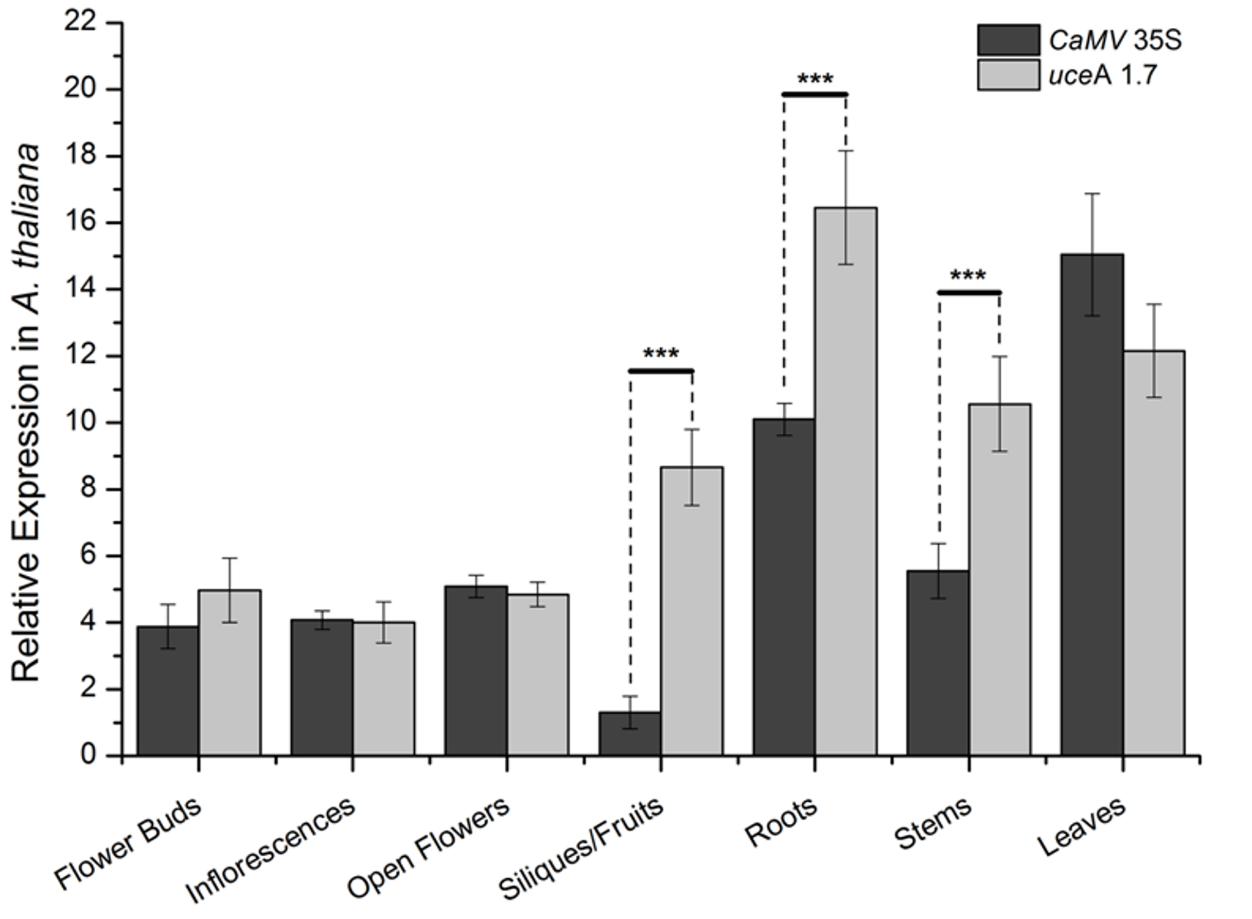  **Figure S3.** **Analyses of candidate promoters supporting the design of the Cry10Aa expression cassette for cotton transformation.** The cotton ubiquitin-conjugate enzyme (*uce*A) constitutive promoter, rather than *CaMV*35S, was used to design the transformation vector. This choice was based on comparative results of the strength and tissue specificity of the *CaMV*35S and *uce*A1.7 promoters, and the *uid*A reporter gene (i.e., GUS-encoding gene) expression levels driven by both promoters was evaluated in GM *A. thaliana*. For this, *uidA* mRNA levels in T_3_ GM *A. thaliana* plants driven by either *CaMV*35S or *uce*A1.7 were measured in several tissues (flower buds, inflorescences, open flowers, siliques/fruits, roots, stems and leaves) through qPCR. Although, the *uidA* mRNA levels driven by the cotton *uce*A1.7 promoter were similar to those driven by the *CaMV*35S promoter in all flower stages, the *uce*A1.7 promoter could drive higher levels of *uidA* transcripts than could the *CaMV*35S promoter in fruits (7-fold) and vegetative tissues, such as the roots and stem (2-fold). These results suggest that the *uce*A1.7 promoter is probably stronger and more specific than *CaMV*35S in cotton fruits, which are the main targets of the CBW. As the aim of this study is cotton protection against CBW, Cry10Aa expression driven by the *uce*A1.7 promoter is probably more appropriate than that driven by the *CaMV*35S promoter in GM cotton. Therefore, the cotton *uce*A1.7 promoter, rather than *CaMV*35S, was used to design the Cry10Aa transformation cassette. The qPCR experimental procedures were as described for Cry10Aa GM cotton plants. The primers used in these experiments are presented in Table S6. The asterisks represent the level of statistical significance between *uid*A expression driven by either the *CaMV*35S or *uce*A1.7 promoter (*Student* *t* test): (*****) *p* ≤ 0.05; (******) 0.05 < *p* ≤ 0.01; (*******) 0.01 < *p* ≤ 0.001. After normalization based on the expression of plant-endogenous genes, the values were plotted relative to the lowest expression value (excluding the WT non-transformed plants) set as expression level 1 (one). |
| --- |
