# Supplementary material for: Transgenic cotton expressing Cry10Aa toxin confers high resistance to the cotton boll weevil
Source: Plant Biotechnol J. 2017 Mar 2;15(8):997–1009. doi: 10.1111/pbi.12694 (PMC5506659; doi:10.1111/pbi.12694)
Supplement: Supplementary file 4 — Figure S4 Phenotypic comparison between T0 Cry10Aa cotton plants and nontransgenic plants. The images compare the macroscopic phenotypes of Cry10Aa cotton P#008 with wild type (WT—BRS 372), both at 4 months of age. In (a) is shown the view of the whole aerial part of transgenic (T) and non‐trangenic (WT) plants. In (b) and (c), the floral structures of WT and P#008 respectively are compared, whereas in (d) and (e) the leaves of the same plants are compared. Both T and WT plants have a medium‐late life cycle, and all vegetative (aerial) and reproductive structures are morphologically similar. This comparison extends to the other T0 plants (P#004, P#005, P#009, P#012, P#014, P#040, P#068, P#082, P#104 and P#128). [file PBI-15-997-s012.docx]

| 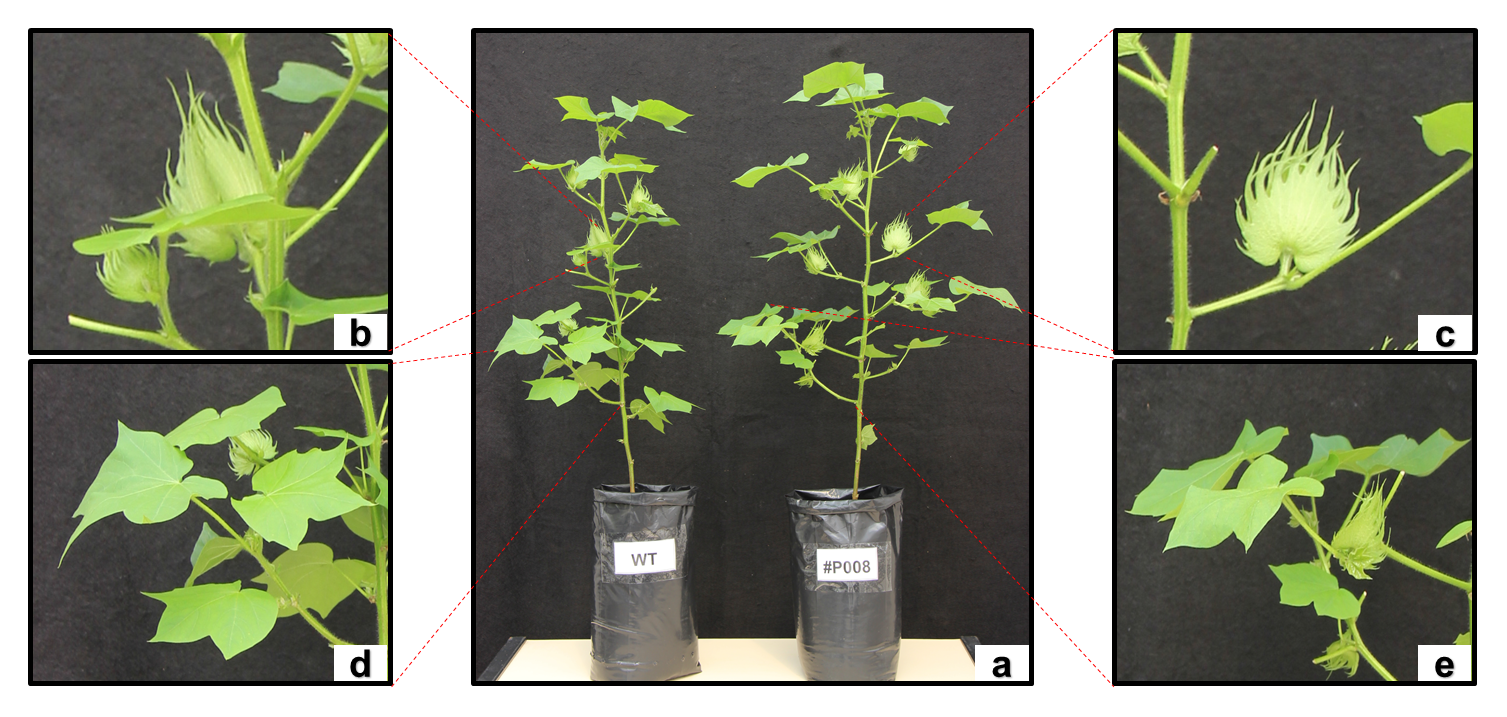  **Figure S4. Phenotypic comparison between T_0_ Cry10Aa cotton plants and non-transgenic plants.** The images compare the macroscopic phenotypes of Cry10Aa cotton P#008 with wildtype (WT - BRS 372) both at four months old. In (**a**) is shown the view of the whole aerial part of WT and transgenic plants. In (**b**) and (**c**), the floral structures of WT and P#008 respectively are compared, whereas in (**d**) and (**e**) the leaves of the same plants are compared. Both WT and transgenic plants have a medium-late life cycle and all vegetative (aerial) and reproductive structures are morphologically similar. This comparison extends to the other T_0_ transformation events (P#004, P#005, P#009, P#012, P#014, P#040, P#068, P#082, P#104 and P#128). |
| --- |
