# Supplementary material for: Transgenic cotton expressing Cry10Aa toxin confers high resistance to the cotton boll weevil
Source: Plant Biotechnol J. 2017 Mar 2;15(8):997–1009. doi: 10.1111/pbi.12694 (PMC5506659; doi:10.1111/pbi.12694)
Supplement: Supplementary file 5 — Figure S5 Leaf area damaged by CBW in T0 GM cotton plants. The graph shows the percentage of leaf area damaged by CBW in leaves of eleven T0 GM cotton plants expressing the toxin Cry10Aa and WT (control), all collected every three days during the bioassays. The rate (damaged leaf area/total leaf area) was obtained using ImageJ software (Schneider et al., 2012). (*) Asterisks represent the level of statistical significance when compared to the damage in WT plants (Student's t‐test): (*) p ≤ 0.05; (**) 0.05 < p ≤ 0.01; (***) 0.01 < p ≤ 0.001. Abbreviation: WT—wild‐type non‐GM plants. [file PBI-15-997-s001.docx]

|   **Figure S5. Leaf area damaged by cotton boll weevil (CBW) in T_0_ GM cotton plants.** The graph shows the percentage of leaf area damaged by CBW in leaves of eleven T_0_ GM cotton plants expressing the toxin Cry10Aa and WT (control), all collected every three days during the bioassays. The rate (damaged leaf area/total leaf area) were obtained using ImageJ software (Schneider et al., 2012). (*) Asterisks represent the level of statistical significance when compared to the damage in WT plants (*Student t* test): (*) *p* ≤ 0.05; (**) 0.05 < *p* ≤ 0.01; (***) 0.01 < *p* ≤ 0.001. **Abbreviation: WT** - wildtype non-GM plants. |
| --- |
