# Supplementary material for: Transgenic cotton expressing Cry10Aa toxin confers high resistance to the cotton boll weevil
Source: Plant Biotechnol J. 2017 Mar 2;15(8):997–1009. doi: 10.1111/pbi.12694 (PMC5506659; doi:10.1111/pbi.12694)
Supplement: Supplementary file 6 — Figure S6 Flower bud drop rate in T0 GM cotton plants infested with CBW. The graph shows the percentage of dropped flower buds in eleven T0 GM cotton plants expressing the toxin Cry10Aa and WT (control) evaluated during the bioassays. (*) Asterisks represent the level of statistical significance when compared to the same rate in WT plants (Student's t‐test): (*) p ≤ 0.05; (**) 0.05 < p ≤ 0.01; (***) 0.01 < p ≤ 0.001. Abbreviation: WT—wild‐type non‐GM plants. [file PBI-15-997-s002.docx]

|   **Figure S6. Flower bud drop rate in T_0_ GM cotton plants infested with cotton boll weevil (CBW).** The graph shows the percentage of dropped flower buds in eleven T_0_ GM cotton plants expressing the toxin Cry10Aa and WT (control) evaluated during the bioassays. (*) Asterisks represent the level of statistical significance when compared to the same rate in WT plants (*Student* *t* test): (*****) *p* ≤ 0.05; (******) 0.05 < *p* ≤ 0.01; (*******) 0.01 < *p* ≤ 0.001. **Abbreviation:** **WT -** wildtype non-GM plants. |
| --- |
