# Supplementary material for: Transgenic cotton expressing Cry10Aa toxin confers high resistance to the cotton boll weevil
Source: Plant Biotechnol J. 2017 Mar 2;15(8):997–1009. doi: 10.1111/pbi.12694 (PMC5506659; doi:10.1111/pbi.12694)
Supplement: Supplementary file 8 — Table S1 LC50 values of recombinant Cry10Aa protein against Anthonomus grandis [file PBI-15-997-s004.docx]

| **Table S1.** LC_50_ values of recombinant Cry10Aa protein against *Anthonomus grandis*^1^ | | | | |
| --- | --- | --- | --- | --- |
| **Sample** | **N** | **Slope ± SE** | **LC_50_ (FL) µg mL^-1^** | **Reference** |
| Cry10Aa protein^2^ | 10 | 2.22 ± 0.24 | 6.35 (5.61 – 7.09) | Present work |
| Cry10Aa protein | 25 | 1.64 ± 0.26 | 7.12 (5.27 – 9.80) | Aguiar et al., 2012 |
| IPS82 *Bt* strain | 25 | 2.64 ± 0.33 | 740 (610 – 910) | Aguiar et al., 2012 |
| S1804 *Bt* strain | 25 | 3.23 ± 0.45 | 300 (250 – 360) | Aguiar et al., 2012 |

^1^*In vitro* bioassay of Cy10Aa toxicity against cotton boll weevil (CBW) larvae: In order to determine the lethal dose that kills 50% of the insect larvae (LC_50_), five doses (0.4, 0.8, 1.6, 6.4 and 12.8 µg mL^-1^ artificial diet) of *E. coli* recombinant Cry10Aa purified protein were added to CBW solid artificial diet according to methodology described by Aguiar et al., 2012 (*Bt Research* **3**, 20-28). Each protein dose was added to 5 mL of the artificial diet before it was poured into six wells plate. Five holes were punched in each diet cylinder (i.e. in each well) and each hole received one CBW neonate larva. The bioassay was kept in an incubator with 14 h photoperiod at 27 ºC. Mortality was recorded seven days later, and the LC_50_ was calculated by Probit analysis (Finney, 1971, *Cambridge University Press, London*).

^2^Heterologous expression of recombinant Cry10Aa toxin in *Escherichia coli*: The *cry10Aa* gene used for heterologous expression in *E. coli* was based in the original sequence of *B. thuringiensis* S1804 strain deposited in the GenBank (Figure S1). The *cry10Aa* gene was amplified by PCR with primers CRY10-*Bam*HI-F and CRY10-*Not*I-R. The amplicon was sub cloned into pET21a expression vector (5443 bp – INVITROGEN^®^, USA) and the resulting pET21a-*cry10Aa* vector (7501 bp) was used to express the gene in *E. coli* BL21(DE3) Star-pLysS The gene expression was induced by adding 0.4 mM IPTG for 24 h and was assessed by 12.0 % SDS/PAGE. The heterologous protein fused with 6-His Tag was purified by affinity chromatography in nickel column (QIAGEN^®^, GERMANY), according to the manufacturer´s instructions.

**Abbreviations in alphabetical order:** *Bt:* *Bacillus thuringiensis*; *FL:* Fiducial limits; *LC:* Lethal concentration); *N:* Number of insects used.
