# Supplementary material for: Transgenic cotton expressing Cry10Aa toxin confers high resistance to the cotton boll weevil
Source: Plant Biotechnol J. 2017 Mar 2;15(8):997–1009. doi: 10.1111/pbi.12694 (PMC5506659; doi:10.1111/pbi.12694)
Supplement: Supplementary file 9 — Table S2 Summary of qPCR experiments—cry10Aa transcript quantification. [file PBI-15-997-s005.docx]

| **Table S2.** Summary of qPCR experiments – *cry10Aa* transcript quantification. | | | | | | |
| --- | --- | --- | --- | --- | --- | --- |
| **Target and endogenous gene cycle threshold (C_t_)**^1^ | | | | | | |
| **Plant ID** | ***cry10Aa***^2^ | | ***Ghubq14***^3^ | | ***Ghpp2a1***^3^ | |
|  | **Leaf** | **Flower Bud** | **Leaf** | **Flower Bud** | **Leaf** | **Flower Bud** |
| **P#004** | 25.40 ± 0.09 | 27.55 ± 0.05 | 20.84 ± 0.05 | 20.85 ± 0.05 | 24.29 ± 0.05 | 24.20 ± 0.02 |
| **P#005** | 25.08 ± 0.20 | 26.54 ± 0.06 | 22.20 ± 0.06 | 21.90 ± 0.04 | 25.32 ± 0.06 | 24.51 ± 0.40 |
| **P#008** | 24.96 ± 0.05 | 26.48 ± 0.03 | 21.90 ± 0.04 | 22.20 ± 0.03 | 25.60 ± 0.08 | 24.73 ± 0.63 |
| **P#009** | 26.43 ± 0.08 | 27.43 ± 0.08 | 21.24 ± 0.05 | 21.19 ± 0.04 | 23.92 ± 0.07 | 24.97 ± 0.69 |
| **P#012** | 26.45 ± 0.09 | 27.45 ± 0.03 | 21.74 ± 0.08 | 21.69 ± 0.04 | 24.18 ± 0.06 | 25.18 ± 0.65 |
| **P#014** | 25.97 ± 0.06 | 26.84 ± 0.17 | 22.14 ± 0.05 | 22.11 ± 0.05 | 25.26 ± 0.06 | 25.38 ± 0.50 |
| **P#040** | 27.56 ± 0.07 | 27.84 ± 0.02 | 20.80 ± 0.14 | 20.99 ± 0.07 | 24.90 ± 0.04 | 25.60 ± 0.08 |
| **P#068** | 27.45 ± 0.07 | 27.68 ± 0.04 | 22.74 ± 0.14 | 22.70 ± 0.12 | 24.79 ± 0.04 | 25.56 ± 0.15 |
| **P#082** | 27.07 ± 0.08 | 27.07 ± 0.08 | 21.71 ± 0.14 | 21.75 ± 0.15 | 24.81 ± 0.05 | 25.50 ± 0.15 |
| **P#104** | 26.81 ± 0.02 | 27.13 ± 0.06 | 22.00 ± 0.12 | 22.20 ± 0.11 | 24.93 ± 0.12 | 25.43 ± 0.13 |
| **P#128** | 26.58 ± 0.09 | 27.51 ± 0.06 | 20.99 ± 0.07 | 20.80 ± 0.14 | 24.03 ± 0.04 | 25.39 ± 0.11 |
| **A. E. (%)**^4^ | 81.74 ± 3.57 | 82.20 ± 4.12 | 83.98 ± 3.67 | 84.89 ± 2.79 | 85.93 ± 4.49 | 86.75 ± 5.35 |

^1^Arithmetic mean and standard error corresponding to all technical and biological replicates.

^2^Target gene.

^3^Endogenous genes.

^4^Amplification efficiency (*cry10Aa* primers: CRY10F and CRY10R; *Ghubq14* primers: UBQ14F and UBQ14R*; Ghpp2a1* primers: PP2A1F and PP2A1R - see Table S1).
