# Supplementary material for: Transgenic cotton expressing Cry10Aa toxin confers high resistance to the cotton boll weevil
Source: Plant Biotechnol J. 2017 Mar 2;15(8):997–1009. doi: 10.1111/pbi.12694 (PMC5506659; doi:10.1111/pbi.12694)
Supplement: Supplementary file 10 — Table S3 Mortality rate (%) of cotton boll weevil adults fed tissues from T0 GM and non‐GM cotton plants. [file PBI-15-997-s013.docx]

| **Table S3.** Mortality rate (%) of cotton boll weevil adults fed on tissues from T_0_ genetically modified (GM) and non-GM control cotton plants. | | | | | |
| --- | --- | --- | --- | --- | --- |
| **Plant ID** | **N**^1^ | **Flower Buds** | **CM**^2^ **(%)** | **Leaves (%)** | **CM**^2^ **(%)** |
| **P#004** | 100 | 50.75 (± 0.00) | 60.00 | 50.13 (± 0.00) | 60.00 |
| **P#005** | 100 | 78.52 (± 10.72) | 87.78 | 83.46 (± 5.77) | 93.33 |
| **P#008** | 100 | 90.75 (± 0.00) | 100.00 | 90.13 (± 0.00) | 100.00 |
| **P#009** | 100 | 54.08 (± 5.77) | 63.33 | 63.46 (± 15.28) | 73.33 |
| **P#012** | 100 | 49.08 (± 11.79) | 58.33 | 52.35 (± 10.72) | 62.22 |
| **P#014** | 100 | 65.19 (± 7.70) | 74.44 | 56.79 (± 5.77) | 66.67 |
| **P#040** | 100 | 54.08 (± 5.77) | 63.33 | 55.407 (± 2.41) | 65.28 |
| **P#068** | 100 | 67.41 (± 5.77) | 76.67 | 74.57 (± 5.09) | 84.44 |
| **P#082** | 100 | 70.75 (± 10.00) | 80.00 | 74.25 (± 1.37) | 84.13 |
| **P#0104** | 100 | 44.08 (± 11.55) | 53.33 | 58.38 (± 2.75) | 68.25 |
| **P#0128** | 100 | 54.08 (± 5.77) | 63.33 | 57.35 (± 7.52) | 67.22 |
| **WT** | 100 | 9.25 (± 1.12) | - | 9.87 (± 1.45) | - |

^1^Number of bioassays.

^2^Correct mortality (CM) estimated by **Schneider-Orelli's formula** (Schneider-Orelli, 1947), $CM \left( \% \right)=\frac{T-C}{100-C} x 100$, where *T* (%) is mortality in treatment and *C* (%) is mortality in control.
