# Supplementary material for: Transgenic cotton expressing Cry10Aa toxin confers high resistance to the cotton boll weevil
Source: Plant Biotechnol J. 2017 Mar 2;15(8):997–1009. doi: 10.1111/pbi.12694 (PMC5506659; doi:10.1111/pbi.12694)
Supplement: Supplementary file 11 — Table S4 Mortality rate (%) of cotton boll weevil adults fed tissues from T1 GM and non‐GM cotton plants. [file PBI-15-997-s007.docx]

| **Table S4.** Mortality rate (%) of cotton boll weevil adults fed on tissues from T_1_ genetically modified (GM) and non-GM control cotton plants. | | | |
| --- | --- | --- | --- |
| **Plant ID** | **N**^1^ | **Leaves** | **CM**^2^ **(%)** |
| **P#004.15** | 100 | 100.00 (± 0.00) | 100.00 |
| **P#005.01** | 100 | 95.00 (± 3.53) | 95.00 |
| **P#005.06** | 100 | 95.00 (± 3.53) | 95.00 |
| **P#008.03** | 100 | 100.00 (± 0.00) | 100.00 |
| **P#008.10** | 100 | 100.00 (± 0.00) | 100.00 |
| **P#008.11** | 100 | 90.00 (± 0.00) | 89.44 |
| **P#009.04** | 100 | 90.00 (± 7.07) | 90.00 |
| **P#014.13** | 100 | 90.00 (± 0.00) | 89.44 |
| **P#082.06** | 100 | 90.00 (± 0.00) | 89.44 |
| **P#082.14** | 100 | 85.00 (± 3.53) | 89.44 |
| **P#104.13** | 100 | 90.00 (± 0.00) | 89.44 |
| **WT** | 100 | 5.00 (± 3.53) | - |

^1^Number of bioassays.

^2^Correct mortality (CM) estimated by **Schneider-Orelli's formula** (Schneider-Orelli, 1947), $CM \left( \% \right)=\frac{T-C}{100-C} x 100$, where *T* (%) is mortality in treatment and *C* (%) is mortality in control.
