# Supplementary material for: Transgenic cotton expressing Cry10Aa toxin confers high resistance to the cotton boll weevil
Source: Plant Biotechnol J. 2017 Mar 2;15(8):997–1009. doi: 10.1111/pbi.12694 (PMC5506659; doi:10.1111/pbi.12694)
Supplement: Supplementary file 12 — Table S5 Commercially approved Bt cotton plants worldwide. [file PBI-15-997-s008.docx]

| **Table S5.**  Commercially approved *Bt* cotton events worldwide. | | | | |
| --- | --- | --- | --- | --- |
| **Event Name and Code** | **Trade Name** | **Gene(s) Introduced** | **Promoter** | **Target Insect** |
| Name: [281-24-236](http://www.isaaa.org/gmapprovaldatabase/event/default.asp?EventID=43&Event=%7brecEvents.EventName%7d) Code: DAS-24236-5 | not available | [cry1F](http://www.isaaa.org/gmapprovaldatabase/gene/default.asp?GeneID=16) | P-4ocsdeltaMas2-SYNTH | Lepdoptera |
| Name: [281-24-236 x 3006-210-23 (MXB-13)](http://www.isaaa.org/gmapprovaldatabase/event/default.asp?EventID=51&Event=%7brecEvents.EventName%7d) Code: DAS-24236-5 x DAS-21Ø23-5 | WideStrike™ Cotton | [cry1Ac](http://www.isaaa.org/gmapprovaldatabase/gene/default.asp?GeneID=17) [cry1F](http://www.isaaa.org/gmapprovaldatabase/gene/default.asp?GeneID=16) | see parentals | Lepdoptera |
| Name: [3006-210-23](http://www.isaaa.org/gmapprovaldatabase/event/default.asp?EventID=44&Event=%7brecEvents.EventName%7d) Code: DAS-21Ø23-5 | not available | [cry1Ac](http://www.isaaa.org/gmapprovaldatabase/gene/default.asp?GeneID=17) | Ubiquitin 1 (*Zea mays*) | Lepdoptera |
| Name: [3006-210-23 x 281-24-236 x MON1445](http://www.isaaa.org/gmapprovaldatabase/event/default.asp?EventID=63&Event=%7brecEvents.EventName%7d) Code: DAS-21Ø23-5 x DAS-24236-5 x MON-Ø1445-2 | WideStrike™ Roundup Ready™ Cotton | [cry1Ac](http://www.isaaa.org/gmapprovaldatabase/gene/default.asp?GeneID=17) [cry1F](http://www.isaaa.org/gmapprovaldatabase/gene/default.asp?GeneID=16) | see parentals | Lepdoptera |
| Name: [3006-210-23 x 281-24-236 x MON88913](http://www.isaaa.org/gmapprovaldatabase/event/default.asp?EventID=64&Event=%7brecEvents.EventName%7d) Code: DAS-21Ø23-5 x DAS-24236-5 x MON-88913-8 | Widestrike™ Roundup Ready Flex™ Cotton | [cry1Ac](http://www.isaaa.org/gmapprovaldatabase/gene/default.asp?GeneID=17) [cry1F](http://www.isaaa.org/gmapprovaldatabase/gene/default.asp?GeneID=16) | see parentals | Lepdoptera |
| Name: [3006-210-23 x 281-24-236 x MON88913 x COT102](http://www.isaaa.org/gmapprovaldatabase/event/default.asp?EventID=374&Event=%7brecEvents.EventName%7d) Code: DAS-21Ø23-5 x DAS-24236-5 x MON-88913-8 x SYN-IR1Ø2-7 | Widestrike™ x Roundup Ready Flex™ x VIPCOT™ Cotton | [cry1Ac](http://www.isaaa.org/gmapprovaldatabase/gene/default.asp?GeneID=17) [cry1F](http://www.isaaa.org/gmapprovaldatabase/gene/default.asp?GeneID=16) [vip3A(a)](http://www.isaaa.org/gmapprovaldatabase/gene/default.asp?GeneID=24) | see parentals | Lepdoptera |
| Name: [31707](http://www.isaaa.org/gmapprovaldatabase/event/default.asp?EventID=257&Event=%7brecEvents.EventName%7d) Code: not available | BXN™ Plus Bollgard™ Cotton | [cry1Ac](http://www.isaaa.org/gmapprovaldatabase/gene/default.asp?GeneID=17) | P-mac (*Agrobacterium tumefaciens* + *CaMV*35S) | Lepdoptera |
| Name: [31803](http://www.isaaa.org/gmapprovaldatabase/event/default.asp?EventID=258&Event=%7brecEvents.EventName%7d) Code: not available | BXN™ Plus Bollgard™ Cotton | [cry1Ac](http://www.isaaa.org/gmapprovaldatabase/gene/default.asp?GeneID=17) | P-mac (*Agrobacterium tumefaciens* + *CaMV*35S) | Lepdoptera |
| Name: [31807 x 31808](http://www.isaaa.org/gmapprovaldatabase/event/default.asp?EventID=52&Event=%7brecEvents.EventName%7d) Code: not available | not available | [cry1Ac](http://www.isaaa.org/gmapprovaldatabase/gene/default.asp?GeneID=17) | see parentals | Lepdoptera |
| Name: [31807](http://www.isaaa.org/gmapprovaldatabase/event/default.asp?EventID=45&Event=%7brecEvents.EventName%7d) Code: not available | BXN™ Plus Bollgard™ Cotton | [cry1Ac](http://www.isaaa.org/gmapprovaldatabase/gene/default.asp?GeneID=17) | P-mac (*Agrobacterium tumefaciens* + *CaMV*35S) | Lepdoptera |
| Name: [31808](http://www.isaaa.org/gmapprovaldatabase/event/default.asp?EventID=46&Event=%7brecEvents.EventName%7d) Code: not available | BXN™ Plus Bollgard™ Cotton | [cry1Ac](http://www.isaaa.org/gmapprovaldatabase/gene/default.asp?GeneID=17) | P-mac (*Agrobacterium tumefaciens* + *CaMV*35S) | Lepdoptera |
| Name: [42317](http://www.isaaa.org/gmapprovaldatabase/event/default.asp?EventID=259&Event=%7brecEvents.EventName%7d) Code: not available | BXN™ Plus Bollgard™ Cotton | [cry1Ac](http://www.isaaa.org/gmapprovaldatabase/gene/default.asp?GeneID=17) | P-mac (*Agrobacterium tumefaciens* + *CaMV*35S) | Lepdoptera |
| Name: [BNLA-601](http://www.isaaa.org/gmapprovaldatabase/event/default.asp?EventID=83&Event=%7brecEvents.EventName%7d) Code: not available | not available | [cry1Ac](http://www.isaaa.org/gmapprovaldatabase/gene/default.asp?GeneID=17) | CaMV35S | Lepidoptera |
| Name: [COT102 (IR102)](http://www.isaaa.org/gmapprovaldatabase/event/default.asp?EventID=74&Event=%7brecEvents.EventName%7d) Code: SYN-IR1Ø2-7 | VIPCOT™ Cotton | [vip3A(a)](http://www.isaaa.org/gmapprovaldatabase/gene/default.asp?GeneID=24) | Actin-2 (*Arabidopsis thaliana*) | Lepdoptera |
| Name: [COT102 x COT67B](http://www.isaaa.org/gmapprovaldatabase/event/default.asp?EventID=76&Event=%7brecEvents.EventName%7d) Code: SYN-IR1Ø2-7 x SYN-IR67B-1 | VIPCOT™ Cotton | [cry1Ab](http://www.isaaa.org/gmapprovaldatabase/gene/default.asp?GeneID=26) [vip3A(a)](http://www.isaaa.org/gmapprovaldatabase/gene/default.asp?GeneID=24) | see parentals | Lepdoptera |
| Name: [COT102 x COT67B x MON88913](http://www.isaaa.org/gmapprovaldatabase/event/default.asp?EventID=77&Event=%7brecEvents.EventName%7d) Code: SYN-IR1Ø2-7 x SYN-IR67B-1 x MON-88913-8 | VIPCOT™ Roundup Ready Flex™ Cotton | [cry1Ab](http://www.isaaa.org/gmapprovaldatabase/gene/default.asp?GeneID=26) [vip3A(a)](http://www.isaaa.org/gmapprovaldatabase/gene/default.asp?GeneID=24) | see parentals | Lepdoptera |
| Name: [COT102 x MON15985](http://www.isaaa.org/gmapprovaldatabase/event/default.asp?EventID=372&Event=%7brecEvents.EventName%7d) Code: SYN-IR1Ø2-7 x MON-15985-7 | Bollgard® III | [cry1Ac](http://www.isaaa.org/gmapprovaldatabase/gene/default.asp?GeneID=17) [cry2Ab2](http://www.isaaa.org/gmapprovaldatabase/gene/default.asp?GeneID=21) [vip3A(a)](http://www.isaaa.org/gmapprovaldatabase/gene/default.asp?GeneID=24) | see parentals | Lepdoptera |
| Name: [COT102 x MON15985 x MON88913](http://www.isaaa.org/gmapprovaldatabase/event/default.asp?EventID=373&Event=%7brecEvents.EventName%7d) Code: SYN-IR1Ø2-7 x MON-15985-7 x MON-88913-8 | Bollgard® III x Roundup Ready™ Flex™ | [cry1Ac](http://www.isaaa.org/gmapprovaldatabase/gene/default.asp?GeneID=17) [cry2Ab2](http://www.isaaa.org/gmapprovaldatabase/gene/default.asp?GeneID=21) [vip3A(a)](http://www.isaaa.org/gmapprovaldatabase/gene/default.asp?GeneID=24) | see parentals | Lepdoptera |
| Name: [COT67B (IR67B)](http://www.isaaa.org/gmapprovaldatabase/event/default.asp?EventID=75&Event=%7brecEvents.EventName%7d) Code: SYN-IR67B-1 | not available | [cry1Ab](http://www.isaaa.org/gmapprovaldatabase/gene/default.asp?GeneID=26) | Actin-2 (*Arabidopsis thaliana*) | Lepdoptera |
| Name: [Event1](http://www.isaaa.org/gmapprovaldatabase/event/default.asp?EventID=79&Event=%7brecEvents.EventName%7d) Code: not available | JK 1 | [cry1Ac](http://www.isaaa.org/gmapprovaldatabase/gene/default.asp?GeneID=17) | *CaMV*35S | Lepdoptera |
| Name: [GFM Cry1A](http://www.isaaa.org/gmapprovaldatabase/event/default.asp?EventID=81&Event=%7brecEvents.EventName%7d) Code: GTL-GFM311-7 | not available | [cry1Ab-Ac](http://www.isaaa.org/gmapprovaldatabase/gene/default.asp?GeneID=30) | *CaMV*35S | Lepdoptera |
| Name: [GHB119](http://www.isaaa.org/gmapprovaldatabase/event/default.asp?EventID=71&Event=%7brecEvents.EventName%7d) Code: BCS-GHØØ5-8 | not available | [cry2Ae](http://www.isaaa.org/gmapprovaldatabase/gene/default.asp?GeneID=23) | *CaMV*35S | Lepdoptera |
| Name: [GHB614 x LLCotton25 x MON15985](http://www.isaaa.org/gmapprovaldatabase/event/default.asp?EventID=73&Event=%7brecEvents.EventName%7d) Code: BCS-GHØØ2-5 x ACS-GHØØ1-3 x MON-15985-7 | not available | [cry1Ac](http://www.isaaa.org/gmapprovaldatabase/gene/default.asp?GeneID=17) [cry2Ab2](http://www.isaaa.org/gmapprovaldatabase/gene/default.asp?GeneID=21) | see parentals | Lepdoptera |
| Name: [GHB614 x MON15985](http://www.isaaa.org/gmapprovaldatabase/event/default.asp?EventID=329&Event=%7brecEvents.EventName%7d) Code: BCS-GHØØ2-5 × MON-15985-7 | not available | [cry1Ac](http://www.isaaa.org/gmapprovaldatabase/gene/default.asp?GeneID=17) [cry2Ab2](http://www.isaaa.org/gmapprovaldatabase/gene/default.asp?GeneID=21) | see parentals | Lepdoptera |
| Name: [GHB614 x T304-40 x GHB119](http://www.isaaa.org/gmapprovaldatabase/event/default.asp?EventID=278&Event=%7brecEvents.EventName%7d) Code: BCS-GHØØ2-5 x BCS-GHØØ4-7 x BCS-GHØØ5-8 | Glytol™ x Twinlink™ | [cry1Ab](http://www.isaaa.org/gmapprovaldatabase/gene/default.asp?GeneID=26) [cry2Ae](http://www.isaaa.org/gmapprovaldatabase/gene/default.asp?GeneID=23) | see parentals | Lepdoptera |
| Name: [GHB614 x T304-40 x GHB119 x COT102](http://www.isaaa.org/gmapprovaldatabase/event/default.asp?EventID=396&Event=%7brecEvents.EventName%7d) Code: BCS-GHØØ2-5 x BCS-GHØØ4-7 x BCS-GHØØ5-8 x SYN-IR1Ø2-7 | Glytol™ x Twinlink™ x VIPCOT™ Cotton | [cry1Ab](http://www.isaaa.org/gmapprovaldatabase/gene/default.asp?GeneID=26) [cry2Ae](http://www.isaaa.org/gmapprovaldatabase/gene/default.asp?GeneID=23) [vip3A(a)](http://www.isaaa.org/gmapprovaldatabase/gene/default.asp?GeneID=24) | see parentals | Lepdoptera |
| Name: [GK12](http://www.isaaa.org/gmapprovaldatabase/event/default.asp?EventID=82&Event=%7brecEvents.EventName%7d) Code: not available | not available | [cry1Ab-Ac](http://www.isaaa.org/gmapprovaldatabase/gene/default.asp?GeneID=30) | *CaMV*35S | Lepdoptera |
| Name: [LLCotton25 x MON15985](http://www.isaaa.org/gmapprovaldatabase/event/default.asp?EventID=66&Event=%7brecEvents.EventName%7d) Code: ACS-GHØØ1-3 x MON-15985-7 | Fibermax™ Liberty Link™ Bollgard II™ | [cry1Ac](http://www.isaaa.org/gmapprovaldatabase/gene/default.asp?GeneID=17) [cry2Ab2](http://www.isaaa.org/gmapprovaldatabase/gene/default.asp?GeneID=21) | see parentals | Lepdoptera |
| Name: [MLS 9124](http://www.isaaa.org/gmapprovaldatabase/event/default.asp?EventID=80&Event=%7brecEvents.EventName%7d) Code: not available | not available | [cry1C](http://www.isaaa.org/gmapprovaldatabase/gene/default.asp?GeneID=29) | *CaMV*35S | Lepdoptera |
| Name: [MON1076](http://www.isaaa.org/gmapprovaldatabase/event/default.asp?EventID=53&Event=%7brecEvents.EventName%7d) Code: MON-89924-2 | Bollgard™ Cotton | [cry1Ac](http://www.isaaa.org/gmapprovaldatabase/gene/default.asp?GeneID=17) | *FMV*35S | Lepdoptera |
| Name: [MON15985](http://www.isaaa.org/gmapprovaldatabase/event/default.asp?EventID=59&Event=%7brecEvents.EventName%7d) Code: MON-15985-7 | Bollgard II™ Cotton | [cry1Ac](http://www.isaaa.org/gmapprovaldatabase/gene/default.asp?GeneID=17) [cry2Ab2](http://www.isaaa.org/gmapprovaldatabase/gene/default.asp?GeneID=21) | *CaMV*35S | Lepdoptera |
| Name: [MON15985 x MON1445](http://www.isaaa.org/gmapprovaldatabase/event/default.asp?EventID=60&Event=%7brecEvents.EventName%7d) Code: MON-15985-7 x MON-Ø1445-2 | Roundup Ready™ Bollgard II™ Cotton | [cry1Ac](http://www.isaaa.org/gmapprovaldatabase/gene/default.asp?GeneID=17) [cry2Ab2](http://www.isaaa.org/gmapprovaldatabase/gene/default.asp?GeneID=21) | see parentals | Lepdoptera |
| Name: [MON531](http://www.isaaa.org/gmapprovaldatabase/event/default.asp?EventID=54&Event=%7brecEvents.EventName%7d) Code: MON-ØØ531-6 | Bollgard™ Cotton, Ingard™ | [cry1Ac](http://www.isaaa.org/gmapprovaldatabase/gene/default.asp?GeneID=17) | *CaMV*35S | Lepdoptera |
| Name: [MON531 x MON1445](http://www.isaaa.org/gmapprovaldatabase/event/default.asp?EventID=61&Event=%7brecEvents.EventName%7d) Code: MON-Ø531-6 x MON-Ø1445-2 | Roundup Ready™ Bollgard™ Cotton | [cry1Ac](http://www.isaaa.org/gmapprovaldatabase/gene/default.asp?GeneID=17) | see parentals | Lepdoptera |
| Name: [MON757](http://www.isaaa.org/gmapprovaldatabase/event/default.asp?EventID=55&Event=%7brecEvents.EventName%7d) Code: MON-ØØ757-7 | Bollgard™ Cotton | [cry1Ac](http://www.isaaa.org/gmapprovaldatabase/gene/default.asp?GeneID=17) | *CaMV*35S | Lepdoptera |
| Name: [MON88701 x MON88913 x MON15985](http://www.isaaa.org/gmapprovaldatabase/event/default.asp?EventID=391&Event=%7brecEvents.EventName%7d) Code: MON 887Ø1-3 x MON-88913-8 x MON-15985-7 | not available | [cry1Ac](http://www.isaaa.org/gmapprovaldatabase/gene/default.asp?GeneID=17) [cry2Ab2](http://www.isaaa.org/gmapprovaldatabase/gene/default.asp?GeneID=21) | see parentals | Lepdoptera |
| Name: [MON88913 x MON15985](http://www.isaaa.org/gmapprovaldatabase/event/default.asp?EventID=62&Event=%7brecEvents.EventName%7d) Code: MON-88913-8 x MON-15985-7 | Roundup Ready™ Flex™ Bollgard II™ Cotton | [cry1Ac](http://www.isaaa.org/gmapprovaldatabase/gene/default.asp?GeneID=17) [cry2Ab2](http://www.isaaa.org/gmapprovaldatabase/gene/default.asp?GeneID=21) | see parentals | Lepdoptera |
| Name: [SGK321](http://www.isaaa.org/gmapprovaldatabase/event/default.asp?EventID=78&Event=%7brecEvents.EventName%7d) Code: not available | not available | [cry1A](http://www.isaaa.org/gmapprovaldatabase/gene/default.asp?GeneID=27)  CpT1* | *CaMV*35S | Lepdoptera |
| Name: [T303-3](http://www.isaaa.org/gmapprovaldatabase/event/default.asp?EventID=68&Event=%7brecEvents.EventName%7d) Code: BCS-GHØØ3-6 | not available | [cry1Ab](http://www.isaaa.org/gmapprovaldatabase/gene/default.asp?GeneID=26) | *CaMV*35S | Lepdoptera |
| Name: [T304-40](http://www.isaaa.org/gmapprovaldatabase/event/default.asp?EventID=69&Event=%7brecEvents.EventName%7d) Code: BCS-GHØØ4-7 | not available | [cry1Ab](http://www.isaaa.org/gmapprovaldatabase/gene/default.asp?GeneID=26) | Ps7s7 (subterranean clover stunt virus) | Lepdoptera |
| Name: [T304-40 x GHB119](http://www.isaaa.org/gmapprovaldatabase/event/default.asp?EventID=72&Event=%7brecEvents.EventName%7d) Code: BCS-GHØØ4-7 x BCS-GHØØ5-8 | TwinLink™ Cotton | [cry1Ab](http://www.isaaa.org/gmapprovaldatabase/gene/default.asp?GeneID=26) [cry2Ae](http://www.isaaa.org/gmapprovaldatabase/gene/default.asp?GeneID=23) | see parentals | Lepdoptera |
| Name: [Silver](http://www.isaaa.org/gmapprovaldatabase/event/default.asp?EventID=72&Event=%7brecEvents.EventName%7d) Six  Code: Ngwe Chi 6 | not available | Unknown *Bt* Protein | not available | Lepdoptera |

Adapted from ISAAA, 2015 (http://www.isaaa.org/gmapprovaldatabase/)
