# Supplementary material for: Transgenic cotton expressing Cry10Aa toxin confers high resistance to the cotton boll weevil
Source: Plant Biotechnol J. 2017 Mar 2;15(8):997–1009. doi: 10.1111/pbi.12694 (PMC5506659; doi:10.1111/pbi.12694)
Supplement: Supplementary file 13 — Table S6 Summary of used primers. [file PBI-15-997-s009.docx]

| **Table S6.** Summary of used primers. | | | | |
| --- | --- | --- | --- | --- |
| **Primer Name** | **Sequence (5’-3’)** | **T_M_ (°C)^1^** | **Amplicon length (bp)** | **Assays** |
|  |  |  |  |  |
| CRY10-*Bam*HI-F | ggatcc ATG CTT GGC GCG TTT GCT GCT CCA GTG^2^ | 58 | 2072 | *cry10Aa* gene cloning in bacteria plasmid vector for heterologous expression. |
| CRY10-*Not*I-R | gcggccgc TTA ATG ATG ATG ATG ATG ATG GCG CAC AAA GAG GTC GTT CAC AAT C^3^ |  |  |  |
|  |  |  |  |  |
| AHASF | GTC ACT GGG TTA ATA TCT CTC GAA TCT TGC A | 57 | 479 | Transformation cassette detection in transgenic cotton plants.  Determination of cassette copy number in transgenic cotton genome. |
| AHASR | CCT ACT TCC AAT GTC TGA TTA GTG CTT CTG G |  |  |  |
|  |  |  |  |  |
| CRY10F | AGT TCA GAA ACA ATA GTC GG | 57 | 410 | Transformation cassette detection in transgenic cotton plants.  Determination of transgene transcript levels in GM cotton plants (RT-qPCR).  Probe synthesis to Southern blot. |
| CRY10R | GCT GGA AGG GTG GGA ATC C |  |  |  |
|  |  |  |  |  |
| GhUBQ14F | CAA CGC TCC ATC TTG TCC TT | 57 | 75 | Determination of transgene transcript levels in GM cotton plants (RT-qPCR). |
| GhUBQ14R | TGA TCG TCT TTC CCG TAA GC |  |  |  |
|  |  |  |  |  |
| GhPP2A1F | GAT CCT TGT GGA GGA GTG GA | 57 | 100 | Determination of transgene transcript levels in GM cotton plants (RT-qPCR). |
| GhPP2A1R | GCG AAA CAG TTC GAC GAG AT |  |  |  |
|  |  |  |  |  |
| GUSF | TGA TAG CGC GTG ACA AAA A | 60 | 92 | Comparison between *CaMV* 35S and *uce*A 1.7 gene promoters (RT-qPCR). |
| GUSR | CGA AAT ATT CCC GTG CAC TT |  |  |  |
|  |  |  |  |  |
| AtHELF | CCA TTC TAC TTT TTG GC GGC T | 60 | 61 | Comparison between *CaMV* 35S and *uce*A 1.7 gene promoters (RT-qPCR). |
| AtHELR | TCA ATG GTA ACT GAT CCA CTC TGA TG |  |  |  |
|  |  |  |  |  |
| AtPP2AF | TAA CGT GGC CAA AAT GAT GC | 60 | 65 | Comparison between *CaMV* 35S and *uce*A 1.7 gene promoters (RT-qPCR). |
| AtPP2AR | GTT CTC CAC AAC CGC TTG GT |  |  |  |
|  |  |  |  |  |
| GhUBC1F | TGG CAT TAT ATT GTC ATT GTT ACT ATC C | 55 | 121 | Determination of cassette copy number in transgenic cotton genome. |
| GhUBC1R | ACC ATG TTA TCT TAT TCT AAG ACA AGC TC |  |  |  |
|  |  |  |  |  |

^1^Anneling (or melting) temperature used in experiments.

^2^Sequence in lowercase (ggatcc) - *Bam*HI restriction enzyme site.

^3^Sequence in lowercase (gcggccgc) - *Not*I restriction enzyme site. The underlined sequence represents 6-His Tag addiction.
